# Supplementary material for: Electrostatically cooperative host-in-host of metal cluster ⊂ ionic organic cages in nanopores for enhanced catalysis
Source: Nat Commun. 2022 Mar 18;13:1471. doi: 10.1038/s41467-022-29031-y (PMC8933400; doi:10.1038/s41467-022-29031-y)
Supplement: Supplementary file 1 — Supplementary Information [file 41467_2022_29031_MOESM1_ESM.pdf]

Supplementary information

Electrostatically cooperative host-in-host of metal cluster ⊂ ionic organic cages in nanopores for enhanced catalysis

Liangxiao Tan[1,2], Jun-Hao Zhou[1], Jian-Ke Sun[1]* & Jiayin Yuan[2]*

[1] MOE Key Laboratory of Cluster Science, Beijing Key Laboratory of Photoelectronic/Electrophotonic Conversion Materials, School of Chemistry and Chemical Engineering, Beijing Institute of Technology, Beijing 102488, P. R. China.

[2] Department of Materials and Environmental Chemistry, Stockholm University, 10691, Stockholm, Sweden

E-mail: jiankesun@bit.edu.cn; jiayin.yuan@mmk.su.se

Table of content

1. Reagents and Characterization 3

2. Synthetic Procedures 4

3. Catalytic Experiments 9

4. Supplementary Figures 13

5. Supplementary Tables 43

6. Computational Details 47

7. Supplementary References 50

1. Reagents and Characterization

All reagents were obtained from chemical companies in high purity and used as received. Fourier-transform infrared (FT-IR) spectra were collected on a Varian 670-IR spectrometer in the wavenumber range of 4000-400 cm^{-1}. ^{1}H and ^{13}C nuclear magnetic resonance (^{1}H-NMR and ^{13}C-NMR) were measured by a Bruker DPX-400 spectrometer operating at 400 MHz and room temperature. ^{13}C CP/MAS solid-state NMR measurement was carried out on a Bruker Avance II WB 400 MHz spectrometer with a contact time of 2 ms and pulse delay of 3 s. Elemental analysis (EA) was performed on a Thermo Flash 2000 Elemental Analyzer. Electrospray ionization mass spectrometry (ESI-MS) was conducted on a Q-TOFs 6520 (Agilent). Powder X-ray diffraction (PXRD) pattern was recorded with a Bruker AXS D8 Advance X-ray diffractometer using Cu-Kα radiation. Thermogravimetric analysis (TGA) was performed on a Discovery TG instrument operating from room temperature to 900 °C with a heating rate of 10 °C min^{-1} in nitrogen atmosphere. N_2 sorption was measured using a Micromeritics ASAP 2020M surface area and porosity analyzer. Before analysis, the samples were degassed at 110 °C for 8 h under vacuum (10^{-5} bar). Pore size distribution was calculated by applying the non-localized density functional theory (NLDFT) model. The field-emission scanning electron microscopy (FE-SEM) images were collected on FEI Nova NanoSEM with an accelerating voltage of 5 kV. The scanning transmission electron microscopy (STEM) images were collected on JEOL JEM-2200FS operated at 200 kV and equipped with a high-angle annular dark-field (HAADF) detector. Inductively coupled plasma optical emission spectrometry (ICP-OES) was performed on the Agilent Varian 700-ES, calibrated with standard solutions. X-ray photoelectron spectroscopy (XPS) analysis was carried out on a ThermoFisher ESCALAB250 X-ray photoelectron spectrometer using an Al-K_{α} source. Zeta potential measurement was conducted on Malvern Zetasizer NanoZS90. UV-Vis absorption spectra were recorded by a Shimadzu UV-1550 spectrometer.

2. Synthetic Procedures

2.1 Synthesis of CC3 porous cage

The CC3 cage molecules were synthesized according to previous literatures. [1,2] Specifically, dichloromethane (10 mL) was added slowly to 1,3,5-triformylbenzene (0.5 g, 3.09 mmol) at room temperature. Then, trifluoroacetic acid (10 μL) was added into the above mixture as a catalyst. Finally, a dichloromethane solution (10 mL) of (R,R)-1,2-diaminocyclohexane (0.53 g, 4.65 mmol) was added. The mixture was capped and left to stand for one week, during which transparent prism-like crystals were formed. The crystalline product was filtrated and washed with dichloromethane/methanol solution (v/v=5/95) for 3 times (50 mL × 3), then vacuum dried at 80 °C for 24 h. The CC3 cage was obtained as white powder (650 mg, yield: 75%).

^{1}H-NMR (400 MHz, $CDCl_3/CD_3OD$): δ 8.17 (s, CH=N, 12H), 7.94 (s, ArH, 12H), 3.40 (m, CH on cyclohexane, 12H), 1.83-1.48 (m, CH_2 on cyclohexane, 48H) ppm; ^{13}C-NMR (400 MHz, $CDCl_3/CD_3OD$): δ 158.88, 135.40, 129.05, 73.52, 31.86, 23.29 ppm; ESI-MS (m/z): $[M+H]^+$ calcd. for $C_{72}H_{84}N_{12}$, 1117.5480; found, 1117.6887; analysis (calcd., found for $C_{72}H_{84}N_{12}$): C (77.38, 77.30), H (7.58, 7.62), N (15.04, 15.00).

2.2 Synthesis of reduced amine CC3 (RCC3) cage

The reduction of CC3 cage was conducted according to previous literatures. [2,3] The CC3 cage (0.5 g, 0.44 mmol) was dissolved in dichloromethane/methanol solution (v/v=1/1, 25 mL) under vigorous stirring. When the solution became transparent, $NaBH_4$ (0.5 g, 13.21 mmol) was directly added into the solution and reacted for 15 h at room temperature. Then, water (1 mL) was introduced and the solution was kept stirring for another 9 h. Finally, the solution was removed under vacuum. The residual was washed with a large amount of water for 3 times (100 mL × 3) to completely remove the decomposed product of $NaBH_4$. If the pH value of the product suspension is higher than 7, the water-washing procedure is continued until it becomes neutral. The resulting sample was then vacuum dried at 80 °C for 24 h to afford RCC3 as white powder (485 mg, yield: 95%).

^{1}H-NMR (400 MHz, CD_3OD): δ 7.13 (s, ArH, 12H), 3.79 (d, $ArCH_2$, 12H), 3.56 (d, $ArCH_2$, 12H), 2.27 (m, CH on cyclohexane, 12H), 2.05-1.07 (m, CH_2 on cyclohexane, 48H) ppm; ^{13}C-NMR (400 MHz, $CDCl_3$): δ 141.06, 125.19, 61.06, 50.31, 31.55, 24.93 ppm; ESI-MS (m/z): $[M+H]^+$ calcd. for $C_{72}H_{108}N_{12}$, 1141.7400; found, 1141.8881; analysis (calcd., found for $C_{72}H_{108}N_{12}$): C (75.74, 75.70), H (9.53, 9.55), N (14.72, 14.66).

2.3 Synthesis of cationic CC3 cage (C-Cage)

The cationic CC3 cage was synthesized by a modified method according to previous literatures, in which the RCC3 cage reacted with hydrochloric acid in water. [2,4] The typical procedure is described as follows: RCC3 cage (1.2 g, 1.06 mmol) was added into water (50 mL), after which concentrated hydrochloric acid (3 mL, 36.00 mmol) was added dropwise at room temperature and the solution was kept stirring for 24 h. Then, the solvent and excess hydrochloric acid were removed by rotary evaporator and the product was vacuum dried at 80 °C for 24 h. C-Cage was obtained as white twinkling powder (1.65 g, yield: 99%).

^{1}H-NMR (400 MHz, D_2O): δ 7.63 (s, ArH, 12H), 4.46 (d, $ArCH_2$, 12H), 4.26 (d, $ArCH_2$, 12H), 3.68 (m, CH on cyclohexane, 12H), 2.35-1.51 (m, CH_2 on cyclohexane, 48H) ppm; ^{13}C-NMR

(400 MHz, D_2O): δ 133.97, 133.45, 58.94, 50.05, 27.18, 23.00 ppm; ESI-MS (m/z): $[M]^+$ calcd. for $C_{72}H_{120}N_{12}^{12+}$, 1153.8360; found, 1153.8796; analysis (calcd., found for $C_{72}H_{120}N_{12}Cl_{12}$): C (54.76, 54.66), H (7.66, 7.58), N (10.64, 10.73).

2.4 Synthesis of CC3-OH porous cage

The CC3-OH cage molecules were synthesized according to previous literature. [5] Specifically, 2-hydroxy-1,3,5-triformylbenzene (71.2 mg, 0.40 mmol) and (R,R)-1,2-diaminocyclohexane (68 mg, 0.60 mmol) were dissolved in DMF solvent (20 mL) and heated at 90 °C for 3 days in a Teflon-lined stainless steel autoclave (50 mL). After slowly cooling to room temperature, the yellow crystalline product was filtrated and washed with acetone for 3 times (50 mL × 3), then vacuum dried at 80 °C for 24 h to afford CC3-OH as orange powder (95 mg, yield 81%).

^1H-NMR (400 MHz, $CDCl_3/CD_3OD$): δ 14.65-13.85 (m, Ar-OH, 4H), 8.60 (m, CH=N, 4H), 8.26 (m, CH=N, 4H) 8.06 (m, CH=N, 4H), 7.83 (m, ArH, 8H), 3.32 (m, CH on cyclohexane, 12H), 1.82-1.25 (m, CH_2 on cyclohexane, 48H) ppm; ^{13}C-NMR (400 MHz, $CDCl_3/CD_3OD$): δ 163.83, 159.57, 155.36, 131.88, 125.67, 123.15, 119.09, 74.21, 32.91, 24.31ppm; ESI-MS (m/z): $[M+H]^+$ calcd. for $C_{72}H_{84}N_{12}O_4$, 1181.5440; found, 1181.6683; analysis (calcd., found for $C_{72}H_{84}N_{12}O_4$): C (73.19, 73.12), H (7.17, 7.14), N (14.23, 14.17).

2.5 Synthesis of reduced CC3-OH anionic cage (A-Cage)

The reduction of CC3-OH cage as well as the anionization were conducted as follows. Specifically, CC3-OH cage (50 mg, 0.042 mmol) was dissolved in dichloromethane/methanol solution (v/v=1/1, 10 mL) under vigorous stirring. When the solution became transparent, $NaBH_4$ (100 mg, 2.63 mmol) was directly added into the solution and reacted for 15 h at room temperature. Then, water (1 mL) was introduced and the solution was kept stirring for another 9 h. Finally, the solution was removed under vacuum. The residual was washed with a large amount of water for 3 times (20 mL × 3) to completely remove the decomposed product of $NaBH_4$. If the pH value of the product suspension is higher than 7, the water-washing procedure is continued until it becomes neutral. The resulting sample was then vacuum dried at 80 °C for 24 h. A-Cage was obtained as pale yellow powder (50 mg, yield: 91%).

^1H-NMR (400 MHz, CD_3OD): δ 6.94 (m, ArH, 8H), 3.73-3.53 (m, $ArCH_2$, 24H), 2.36 (m, CH on cyclohexane, 12H), 2.17-1.09 (m, CH_2 on cyclohexane, 48H) ppm; ^{13}C-NMR (400 MHz, CD_3OD): δ 156.66, 132.89, 129.65, 61.84, 50.66, 31.94, 25.90 ppm; ESI-MS (m/z): $[M+2H]^-$ calcd. for $C_{72}H_{106}N_{12}O_4^{2-}$, 1203.7200; found, 1203.8463; analysis (calcd., found for $C_{72}H_{104}N_{12}O_4Na_4$): C (66.85, 66.79), H (8.10, 8.11), N (12.99, 13.07).

2.6 Synthesis of ion pair-directed supramolecular assemblies (C-Cage-SS, C-Cage-VB and A-Cage-imidaz)

The supramolecular assemblies were obtained by ion exchange of ionic cage with the reactive vinyl monomer salts, in which the sodium styrene sulfonate (SS) and sodium 4-vinylbenzonic acid (VB) were selected for cationic C-Cage and 1-cyanomethyl-3-vinylimidazolium bromide (imidaz) was selected for anionic A-Cage. By changing ion exchange ratio, the ionic monomer amount in resulting supramolecular assembly can be easily adjusted. For C-Cage-SS-Y (Y stand for the molar ratio of SS to C-Cage), the molar ratios were varied from 12:1 to 9:1 and 6:1 (SS to C-Cage ratio); however, the molar ratio was fixed at 12:1 for

C-Cage-VB (VB to C-Cage) and 4:1 for A-Cage-imidaz (imidaz to A-Cage).

Specifically, certain amount of monomer (24.7 mg, 0.12 mmol; 18.5 mg, 0.09 mmol; 12.4 mg, 0.06 mmol for SS; 20.4 mg, 0.12 mmol for VB and 8.6 mg, 0.04 mmol for imidaz respectively) dissolved in water (1 mL) was directly added into the ionic cage solution (15.8 mg, 0.01 mmol in 10 mL of water for C-Cage and 12.9 mg, 0.01 mmol in 5 mL of methanol for A-Cage). The supramolecular assemblies were formed immediately as precipitation which were further centrifuged and washed with water for 3 times (10 mL × 3) subsequently. The resulting products were freeze dried for 24 h to afford C-Cage-SS-12 (32.3 mg, yield: 96%), C-Cage-SS-9 (27.5 mg, yield: 95%), C-Cage-SS-6 (23.2 mg, yield: 94%), C-Cage-VB (27.8 mg, yield: 95%) as white powder and A-Cage-imidaz (16.5 mg, yield: 95%) as pale yellow powder.

C-Cage-SS-12:

^{1}H-NMR (400 MHz, CD_3OD): δ 7.90 (s, ArH from C-Cage, 12H), 7.82 (d, ArH from SS, 24H), 7.52 (d, ArH from SS, 24H), 6.78 (dd, Ar-CH from SS, 12H), 5.89 (d,=CH_2 from SS, 12H), 5.34 (d,=CH_2 from SS, 12H), 4.32-4.26 (m, ArCH_2 from C-Cage, 24H), 3.75 (m, CH on cyclohexane from C-Cage, 12H), 2.30-1.38 (m, CH_2 on cyclohexane from C-Cage, 48H) ppm; ^{13}C-NMR (400 MHz, CD_3OD): δ 144.94, 141.33, 137.09, 134.64, 133.03, 127.44, 116.42, 58.32, 49.73, 27.00, 22.91 ppm; ESI-MS (m/z): $[M]^+$ calcd. for $C_{72}H_{120}N_{12}^{12+}$, 1153.8360; found, 1153.8796; analysis (calcd., found for $C_{168}H_{204}N_{12}S_{12}O_{36}$): C (60.19, 60.08), H (6.13, 6.05), N (5.01, 5.17).

C-Cage-SS-9:

^{1}H-NMR (400 MHz, CD_3OD): δ 7.85 (s, ArH from C-Cage, 12H), 7.83 (d, ArH from SS, 18H), 7.49 (d, ArH from SS, 18H), 6.76 (dd, Ar-CH from SS, 9H), 5.87 (d,=CH_2 from SS, 9H), 5.35 (d,=CH_2 from SS, 9H), 4.34-4.18 (m, ArCH_2 from C-Cage, 24H), 3.54 (m, CH on cyclohexane from C-Cage, 12H), 2.17-1.31 (m, CH_2 on cyclohexane from C-Cage, 48H) ppm; ESI-MS (m/z): $[M]^+$ calcd. for $C_{72}H_{120}N_{12}^{12+}$, 1153.8360; found, 1153.8796; analysis (calcd., found for $C_{144}H_{183}N_{12}S_9O_{27}Cl_3$): C (59.46, 59.40), H (6.34, 6.25), N (5.78, 5.80).

C-Cage-SS-6:

^{1}H-NMR (400 MHz, CD_3OD): δ 7.92 (s, ArH from C-Cage, 12H), 7.86 (d, ArH from SS, 12H), 7.54 (d, ArH from SS, 12H), 6.77 (dd, Ar-CH from SS, 6H), 5.91 (d,=CH_2 from SS, 6H), 5.36 (d,=CH_2 from SS, 6H), 4.32 (m, ArCH_2 from C-Cage, 24H), 3.77 (m, CH on cyclohexane from C-Cage, 12H), 2.34-1.43 (m, CH_2 on cyclohexane from C-Cage, 48H) ppm; ESI-MS (m/z): $[M]^+$ calcd. for $C_{72}H_{120}N_{12}^{12+}$, 1153.8360; found, 1153.8796; analysis (calcd., found for $C_{120}H_{162}N_{12}S_6O_{18}Cl_6$): C (58.45, 58.39), H (6.62, 6.50), N (6.82, 6.93).

C-Cage-VB:

^{1}H-NMR (400 MHz, CD_3OD): δ 7.83 (d, ArH from VB, 24H), 7.60 (s, ArH from C-Cage, 12H), 7.52 (d, ArH from VB, 24H), 6.77 (dd, Ar-CH from VB, 12H), 5.90 (d,=CH_2 from VB, 12H), 5.36 (d,=CH_2 from VB, 12H), 4.10-3.92 (m, ArCH_2 from C-Cage, 24H), 2.94 (m, CH on cyclohexane from C-Cage, 12H), 2.08-1.24 (m, CH_2 on cyclohexane from C-Cage, 48H) ppm; ^{13}C-NMR (400 MHz, d_6-DMSO): δ 166.55, 146.29, 137.83, 135.96, 132.66, 131.71, 125.71, 115.23, 55.61, 47.75, 25.24, 21.27 ppm; ESI-MS (m/z): $[M]^+$ calcd. for $C_{72}H_{120}N_{12}^{12+}$, 1153.8360; found, 1153.8796; analysis (calcd., found for $C_{180}H_{204}N_{12}O_{24}$): C (74.05, 73.91), H (7.04, 7.15), N (5.76, 5.57).

A-Cage-imidaz:

^{1}H-NMR (400 MHz, CD_3OD): δ 8.07 (m, N-CH from imidaz, 4H),7.76 (m, N-CH from imidaz, 4H), 7.28 (dd, -CH= from imidaz, 4H), 6.96 (m, ArH from A-Cage, 8H), 5.94 (d,=CH_2 from imidaz, 4H), 5.49 (d,=CH_2 from imidaz, 4H), 3.83-3.51 (m, $ArCH_2$ from A-Cage, 24H), 2.34 (m, CH on cyclohexane from A-Cage, 12H), 2.15-1.08 (m, CH_2 on cyclohexane from A-Cage, 48H) ppm; ^{13}C-NMR (400 MHz, CD_3OD): δ 155.10, 137.25, 131.28, 129.76, 128.40, 125.20, 120.95, 110.52, 61.09, 51.02, 31.61, 26.09 ppm; ESI-MS (m/z): $[M]^-$ calcd. for $C_{72}H_{104}N_{12}O_4^{4-}$, 1201.7040; found, 1201.8691; analysis (calcd., found for $C_{100}H_{136}N_{24}O_4$): C (69.09, 69.09), H (7.89, 7.94), N (19.34, 19.42).

2.7 Synthesis of hierarchical host-in-host porous cage-hyper-crosslinked porous poly(ionic liquid) composites (C-Cage$^+$⊂PoPIL$^-$ and A-Cage$^-$⊂PoPIL$^+$)

The porous polymer composites were prepared by direct radical polymerization of the supramolecular assembly with divinylbenzene (DVB) crosslinker (Supplementary Figure 1-3). Typically, the supramolecular assembly (100 mg) was dissolved in DMSO solvent (20 mL) with DVB crosslinker (100 mg, 0.77 mmol) and AIBN initiator (10 mg, 5 wt% of total monomer). The mixture was deoxygenated 3 times by a freeze-pump-thaw procedure and charged with nitrogen, then placed in an oil bath and reacted at 65 °C for 24 hours. After cooling down to room temperature, the mixture was filtrated and washed with ethanol for 3 times (50 mL × 3). Finally, the resulting product was vacuum dried at 80 °C for 24 h to afford the porous composite as white powder. The yields for all of the composites are more than 99% due to the high efficiency of the co-polymerization. The obtained polymer composites were named as C-Cage$^+$⊂PoPIL$^-$-Y and A-Cage$^-$⊂PoPIL$^+$-Y according to the inner cage host, where Y represents the ion exchange molar ratio in the supramolecular assembly. The porous structure in the resulting polymer composites were modulated by varying the DVB crosslinker amount during the polymerization, in which the mass ratio of DVB to C-Cage-SS was tuned from 1:1 to 3:1 and 9:1.

2.8 Synthesis of Au⊂C-Cage$^+$ and their porous polymer composites ([Au⊂C-Cage$^+$]⊂PoPIL$^-$)

The encapsulation of metal clusters in cationic CC3 cage was conducted according to previous literature. [2] Here, Au cluster was introduced as a representative catalytic active site. In a typical synthesis, C-Cage (15.8 mg, 0.01 mmol) dissolved in water (9 mL) was added by an aqueous solution containing $H_3AuCl_4 \cdot 3H_2O$ (0.5 mL, 0.5 mg Au in content). After aging for 5 mins, the homogeneous mixture was added by a $NaBH_4$ solution (0.5 mL, 4 mg/mL) with vigorously shaking which resulted a well transparent dispersion of Au⊂C-Cage$^+$ solution. The ion exchange of the Au⊂C-Cage$^+$ and polymerization were the same to afore-mentioned procedures.

The supramolecular assemblies of Au⊂C-Cage-SS-Y were obtained as light-yellow powder with similar yield as C-Cage-SS-Y (32.6 mg, yield: 96% for Au⊂C-Cage-SS-12, 27.9 mg, yield: 94% for Au⊂C-Cage-SS-9 and 23.8 mg, yield: 95% for Au⊂C-Cage-SS-6). And their corresponding composites [Au⊂C-Cage$^+$]⊂PoPIL$^-$-Y were obtained as white powder with similar yields as C-Cage$^+$⊂PoPIL$^-$ (all yields > 99%).

2.9 Synthesis of Au nanoparticle embedded catalyst (Au⊂PoPIL$^-$)

The Au⊂PoPIL$^-$ composite catalyst was synthesized by post immobilization of Au

nanoparticle in the porous polymer composite (PoPIL^{-}), however, without inner C-Cage host. First, the composite was prepared by directly radical polymerization of SS monomer (73.7 mg, 0.358 mmol) and DVB crosslinker (100 mg, 0.77 mmol) with the same monomer content to that of C-Cage^{+}⊂PoPIL^{-} (AIBN was used as the initiator, 8.7 mg 5wt%). The experimental detail was also similar and generated PoPIL^{-} as white powder but with a relative lower yield (140 mg, yield: 80%). Then, the post immobilization process was realized by an in-situ reduction of Au precursor in PoPIL^{-}. Briefly, 10 mg of PoPIL^{-} was dispersed in a $H_3AuCl_4 \cdot 3H_2O$ containing aqueous solution (2 mL, 2.5 mM) and stirred for 30 mins, then the dispersion was centrifuged, and the residual was immersed in a $NaBH_4$ solution (10 mM, 1 mL) to generate Au nanoparticles in porous polymer matrix. The resulting composite was named as Au⊂PoPIL^{-}.

3. Catalytic Experiments

To eliminate the catalytic activity discrepancy caused by temperature, all the following catalytic experiments were conducted at 298 K.

3.1 Charge selective catalytic reaction

3.1.1 Catalytic performance comparison

The [Au⊂C-Cage$^+$]⊂PoPIL$^-$ (with SS as the counteranion and molar ratio of SS to C-Cage is 12:1, mass ratio of C-Cage-SS to DVB is 1:1 if without special instruction) was selected as the typical heterogeneous catalyst to evaluate the catalytic performance. Model substrates with similar molecule weight and charge property were utilized including methylene blue (MB, Mw 320, positively charged) and methyl orange (MO, Mw 327, negatively charged). Another kind of organic micropollutant 2-amino 5-nitro pyridinium chloride (2A5NPCl, Mw 175.5, positively charged) was also selected to illustrate the versatility of current catalyst (Supplementary Figure 48).

In a typical experiment, [Au⊂C-Cage$^+$]⊂PoPIL$^-$ (2 mg) was added into the certain substrate solution (1 mg/mL, 10 mL). After 30 mins equilibrium of adsorption in dark, $NaBH_4$ (3.8 mg, 0.1 mmol) was added as the reducing agent. The adsorption of substrate as well as the catalytic reaction process was monitored by UV-Vis spectroscopy. In comparison, the nonconfined Au⊂C-Cage$^+$ catalyst (0.645 mg, calculated according to the ICP results, in Supplementary Table 4) with the same amount of Au as [Au⊂C-Cage$^+$]⊂PoPIL$^-$ catalyst was utilized.

The catalytic process was fitted by the first-order kinetic model as expressed by the following equation:

$$\text{Supplementary Equation (1): } -\ln\left(\frac{C}{C_0}\right) = kt$$

The C_0 and C are the initial and current concentration of the substrate, respectively, and k is the kinetic rate constant.

3.1.2 Coulombic effect

To evaluate the Coulombic effect for the [Au⊂C-Cage$^+$]⊂PoPIL$^-$ catalyst in the charge selective catalytic reaction, Debye screening length and surface potential were modulated by altering the ionic strength in solution and the charge density on the outer shell, respectively. First, the ionic strength was enhanced by applying the MB degradation catalytic reaction in the 1X phosphate-buffered saline solution (PBS, composed of 137 mM NaCl, 2.7 mM KCl, 10 mM Na_2HPO_4 and 1.8 mM KH_2PO_4) in which the other reaction conditions were the same as those of the catalytic reaction in water media. Specifically, [Au⊂C-Cage$^+$]⊂PoPIL$^-$ (2 mg) was added into the MB at 1X PBS solution (1 mg/mL, 10 mL). After 30 mins equilibrium of adsorption in dark, $NaBH_4$ (3.8 mg, 0.1 mmol) was added as the reducing agent. The catalytic reaction process was monitored by UV-Vis spectroscopy.

Then, the influence of charge density on the outer shell of the [Au⊂C-Cage$^+$]⊂PoPIL$^-$ catalyst was investigated by using different catalysts with pre-adjusted anion exchange ratio, *i.e.*, regulating the initial mixing molar ratio between SS anion and Au⊂C-Cage$^+$ (from 12:1 to 9:1 and 6:1 in the anion exchange step prior to polymerization). Specifically, different [Au⊂C-Cage$^+$]⊂PoPIL$^-$-Y catalysts (2 mg, Y=12, 9 and 6) were added into the MB at water solution (1 mg/mL, 10 mL), respectively. After 30 mins equilibrium of adsorption in dark,

$NaBH_4$ (3.8 mg, 0.1 mmol) was added as the reducing agent. The catalytic reaction process was monitored by UV-Vis spectroscopy.
The catalytic process was fitted by the first-order kinetic model as expressed by the following equation:

$$\text{Supplementary Equation (2): } -\ln\left(\frac{C}{C_0}\right) = kt$$

The C_0 and C are the initial and current concentration of the substrate, respectively, and k is the kinetic rate constant.

3.1.3 Reusability of the catalyst
The reusability of the catalyst was evaluated by applying the MB decomposition reaction for 5 successive cycles. In detail, the heterogeneous [Au⊂C-Cage$^+$]⊂PoPIL$^-$ catalyst (2 mg) was separated by centrifuge after the first run and washed with water for 3 times (10 mL × 3), then directly added into another MB solution with the same concentration (1 mg/mL, 10 mL) and kept stirring in dark for 30 mins to fulfil the adsorption equilibrium. After which, $NaBH_4$ (3.8 mg, 0.1 mmol) was added and the catalytic reaction process was monitored by UV-Vis spectroscopy. The catalytic reaction was tested for 5 cycles in total.

3.1.4 Charge selective competition catalysis
The selectivity of the [Au⊂C-Cage$^+$]⊂PoPIL$^-$ catalyst was investigated by applying the competitive decomposition of positive MB and negative MO substrates with equal amount in solution. Specifically, [Au⊂C-Cage$^+$]⊂PoPIL$^-$ (2 mg) was added into the dual substrates solution (1 mg/mL for both MB and MO substrates, 10 mL). After 30 mins equilibrium of adsorption in dark, $NaBH_4$ (3.8 mg, 0.1 mmol) was added as the reducing agent. The catalytic reaction process was monitored by UV-Vis spectroscopy.

3.2 Enzymatic-like cascade reaction
3.2.1 Preparation of cationic ferrocene catalyst (Fer$^+$)
The cationic ferrocene catalyst was prepared by cationization of a neutral ferrocene precursor. Specifically, (dimethylaminomethyl)ferrocene (243 mg, 1 mmol) was added into water (10 mL) as well as concentrated hydrochloric acid (0.1 mL, 1.2 mmol) at room temperature. The mixture was kept stirring for 24 h, then water and excess hydrochloric acid was removed under vacuum at 80 °C for 24 h to afford the resulting Fer$^+$ product as dark orange powder (275 mg, yield: 98%).
^{1}H-NMR (400 MHz, D_2O): δ 4.53-4.37 (m, H on ferrocene, 9H), 4.12 (s, $-CH_2-$, 2H), 2.76 (s, $-CH_3$, 6H) ppm; ^{13}C-NMR (400 MHz, D_2O): δ 74.13, 71.23, 70.30, 69.32, 57.57, 41.53 ppm; ESI-MS (m/z): $[M]^+$ calcd. for $C_{13}H_{18}FeN^+$, 244.1390; found, 244.0779; analysis (calcd., found for $C_{13}H_{18}FeNCl$): C (55.85, 55.93), H (6.49, 6.48), N (5.01, 5.03).

3.2.2 Encapsulation of Fer$^+$ catalyst
Working curve of the Fer$^+$
Certain amount of Fer$^+$ was dissolved in water to generate solution with various concentration of 0.25 mM, 0.5 mM, 1 mM, 2 mM, 5 mM and 10 mM. The absorbance was recorded by UV-Vis spectroscopy and fitted by a linear equation.

Typical encapsulation procedure

[Au⊂C-Cage$^+$]⊂PoPIL$^-$ (2 mg) was soaked in the Fer$^+$ solution (0.5 mL, 2 mM) and kept stirring at room temperature for 1 h. Then, the suspension was centrifuged and directly dried at 80 °C for 24 h under vacuum to afford the [Au⊂C-Cage$^+$&Fer$^+$]⊂PoPIL$^-$ catalyst. The encapsulation amount was calculated by comparing the initial and final concentration of Fer$^+$ solution using the UV-Vis spectroscopy. The successful encapsulation was further confirmed by elemental mapping, XPS and ICP-OES.

3.2.3 Activity evaluation of the enzymatic-like TMB oxidation cascade reaction

Initial reaction velocity comparison

The enzymatic-like cascade reaction activity was evaluated by the steady-state kinetic assays, according to the procedures published in literature. [6] Specifically, all the assays were conducted in a cuvette (path length, l =1.0 cm) with 1 M glucose solution. In a typical assay, [Au⊂C-Cage$^+$&Fer$^+$]⊂PoPIL$^-$ (2mg) was first dispersed in 2 mL glucose solution (1 M) with 3,3′,5,5′-tetramethylbenzidine (TMB) solution (16 mM, 20 μL). Then, the reaction was initiated by inletting oxygen. The absorbance of the reaction solution at λ_{max}=652 nm was afterwards detected by the UV-vis spectroscopy with the interval of 10 mins and lasted for 2 h. In comparison, a mixture of free Au⊂C-Cage$^+$ (0.612 mg) and Fer$^+$ (50 mM, 3.14 μL, calculated according to the ICP results, in Supplementary Table 4) catalysts with the same Au and Fe content as [Au⊂C-Cage$^+$&Fer$^+$]⊂PoPIL$^-$ catalyst were also evaluated.

The absorbance versus time curve was used to derive the initial reaction velocity (v) through the following equation:

$$\text{Supplementary Equation (3):}\ v = \frac{Slope_{initial}}{\varepsilon_{TMB} \times l}$$

Herein, $Slope_{initial}$ is the first derivation from the initial point on the measured curve, ε_{TMB} is the molar extinction coefficient of oxTMB (3.9×10^4 $M^{-1}\cdot cm^{-1}$, according to the published literature [6]) and l is the path length (1.0 cm).

H_2O_2 generation detection

The H_2O_2 generated by Au catalyzed oxidation of glucose was confirmed and quantified by a spectrophotometry with titanium oxalate in acidic solution. Specifically, a mixture of free Au⊂C-Cage$^+$ (0.612 mg) and Fer$^+$ (50 mM, 3.14 μL) as well as confined [Au⊂C-Cage$^+$&Fer$^+$]⊂PoPIL$^-$ (2 mg) were added into 1 M glucose solution (2 mL), respectively. Then, oxygen was bubbled to initialize the oxidation of glucose. After 2 h, the H_2O_2 containing solutions were centrifugated and the supernatants were added with titanium oxalate (0.5 mL, 0.1 M) and sulfuric acid (0.5 mL, 0.1 M), shaking for 5 mins and analyzed by UV-Vis spectroscopy. The H_2O_2 concentration was obtained by fitting the absorbance value at 385 nm with the working curve.

K_m calculation using the Michaelis-Menten equation

K_m, representing the affinity between substrate and catalyst, was obtained by using the Michaelis-Menten equation, in which the initial reaction velocity of TMB oxidation with different TMB concentration was evaluated. In these assays, a mixture of free Au⊂C-Cage$^+$ (0.612 mg) and Fer$^+$ (50 mM, 3.14 μL) as well as confined [Au⊂C-Cage$^+$&Fer$^+$]⊂PoPIL$^-$ (2 mg) were used as catalysts, the TMB concentration varied from 0.1 to 0.5 mM (2 mL), and the H_2O_2 concentration was fixed at 10 mM (1M, 20 μL). The absorbance of the reaction

solution at λ_{max}=652 nm was detected by the UV-vis spectroscopy with the interval of 5 mins and lasted for 30 mins to calculate the initial reaction velocity. Then the plot of initial reaction velocity (v) against TMB concentration can be obtained and fitted by the nonlinear regression of the Michaelis-Menten equation:

$$\text{Supplementary Equation (4): } v = V_{max} \times \frac{[S]}{K_m+[S]}$$

where *Vmax* is the maximal reaction velocity, *[S]* is the concentration of the TMB and *Km* is the Michaelis constant. The values of *Km* and *Vmax* can be obtained from the double reciprocal plots.

Substrate channelling investigation

The substrate channelling effect was confirmed by adding a competing reagent which can react with the intermediate H_2O_2 (Supplementary Figure 58). Here, thiourea was selected as an organic reducing agent to participate in the cascade reaction. Briefly, [Au⊂C-Cage$^+$&Fer$^+$]⊂PoPIL$^-$ (2 mg) was added into 1 M glucose solution (2 mL) with TMB solution (16 mM, 20 μL) and a certain amount of thiourea (0.1 M, 10-40 μL). Then, oxygen was bubbled to initialize the reaction and the catalytic process was monitored by measuring the absorbance of the reaction solution at λ_{max}=652 nm using the UV-vis spectroscopy. Finally, the initial reaction velocity (v) against competing reagent amount can be obtained. In comparison, a mixture of free Au⊂C-Cage$^+$ (0.612 mg) and Fer$^+$ (50 mM, 3.14 μL) catalysts with the same Au and Fe content as [Au⊂C-Cage$^+$&Fer$^+$]⊂PoPIL$^-$ catalyst were also evaluated.

3.2.4 Activity evaluation of the enzymatic-like OPD oxidation cascade reaction

The cascade catalytic oxidation of o-phenylenediamine (OPD) was similar to that of the TMB oxidation. Specifically, [Au⊂C-Cage$^+$&Fer$^+$]⊂PoPIL$^-$ (2mg) was dispersed in 2 mL of glucose solution (1 M) with of OPD solution (50 mM, 50 μL) in a cuvette (path length, l =1.0 cm). Then, the reaction was initiated by inletting oxygen. The absorbance of the reaction solution at λ_{max}=447 nm was afterwards detected by the UV-vis spectroscopy with the interval of 5 min and lasted for 1 h. In comparison, a mixture of free Au⊂C-Cage$^+$ (0.612 mg) and Fer$^+$ (50 mM, 3.14 μL) catalysts with the same Au and Fe content as [Au⊂C-Cage$^+$&Fer$^+$]⊂PoPIL$^-$ catalyst were also evaluated.

The absorbance versus time curve was used to derive the initial reaction velocity (v) through the following equation:

$$\text{Supplementary Equation (5): } v = \frac{Slope_{initial}}{\varepsilon_{OPD} \times l}$$

Herein, $Slope_{initial}$ is the first derivation from the initial point on the measured curve, ε_{OPD} is the molar extinction coefficient of 2,3-diaminophenazine (oxOPD) product (2.1×10^4 $M^{-1}\cdot cm^{-1}$, according to the published literature[7]) and l is the path length (1.0 cm).

4. Supplementary Figures

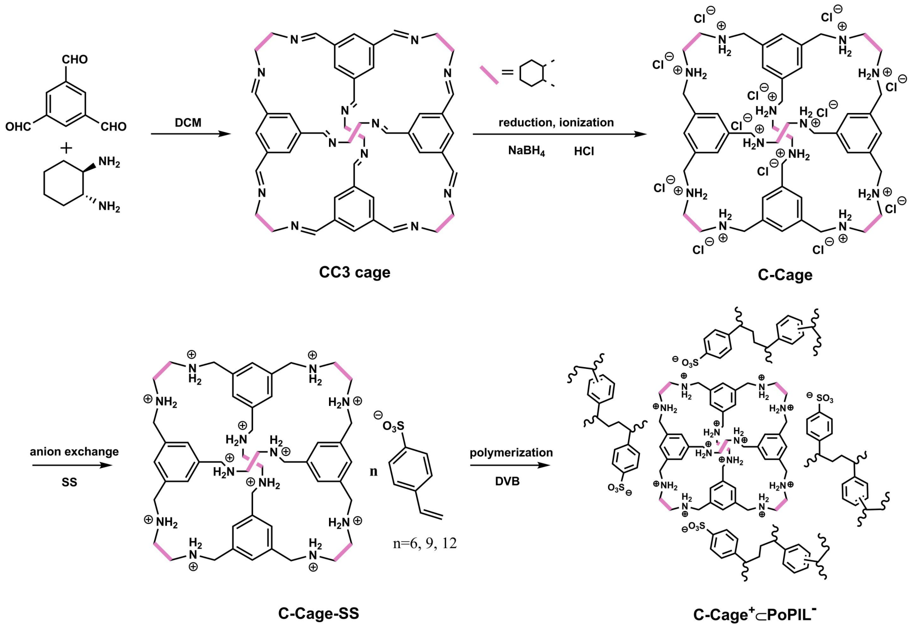

Supplementary Figure 1. Synthetic procedure for the C-Cage$^+$⊂PoPIL$^-$ with SS counteranion (a portion of chloride ions remained to keep the C-Cage-SS supramolecular assembly neutral when the anion exchange ratio is 6:1 or 9:1).

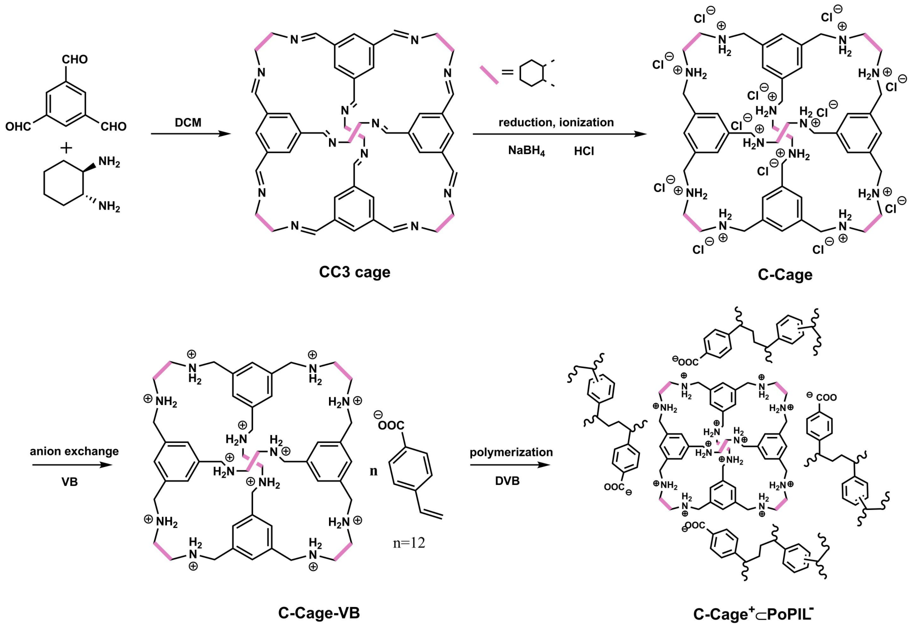

Supplementary Figure 2. Synthetic procedure for the C-Cage$^+$⊂PoPIL$^-$ with VB counteranion.

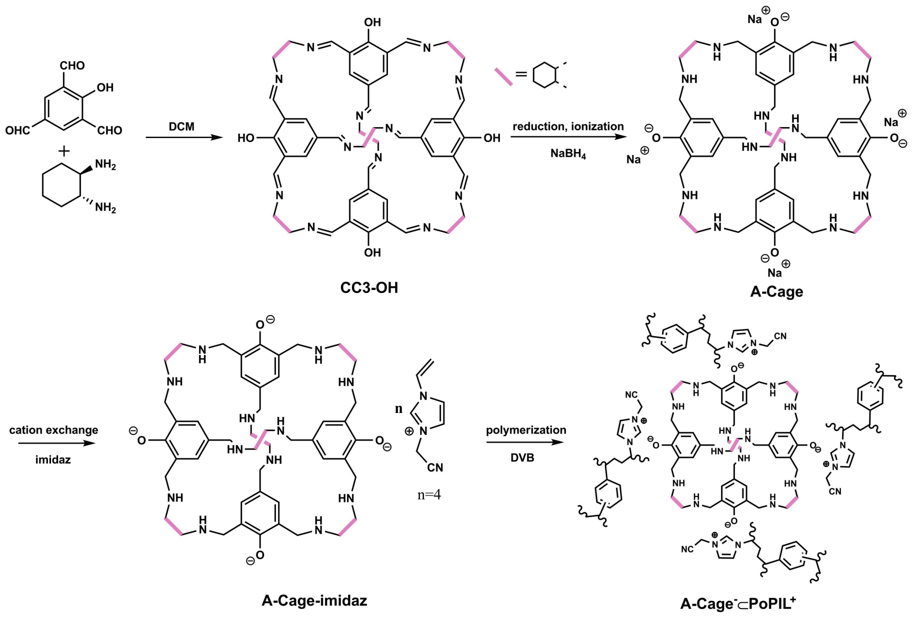

Supplementary Figure 3. Synthetic procedure for the A-Cage$^-$⊂PoPIL$^+$ with imidaz countercation.

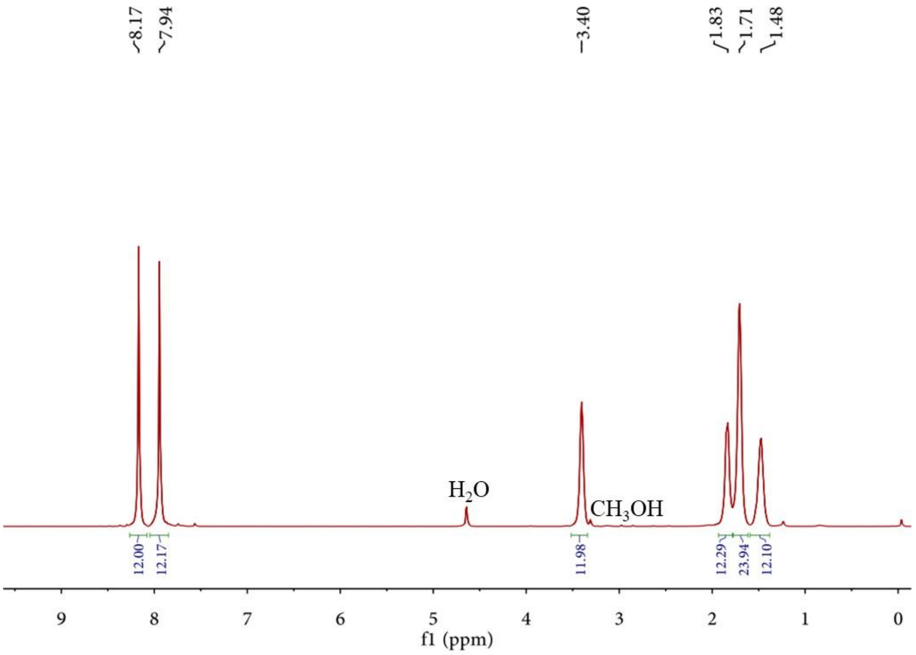

Supplementary Figure 4. ^{1}H-NMR spectrum of CC3 cage in $CD_3OD/CDCl_3$ (1:1) mixture solvent.

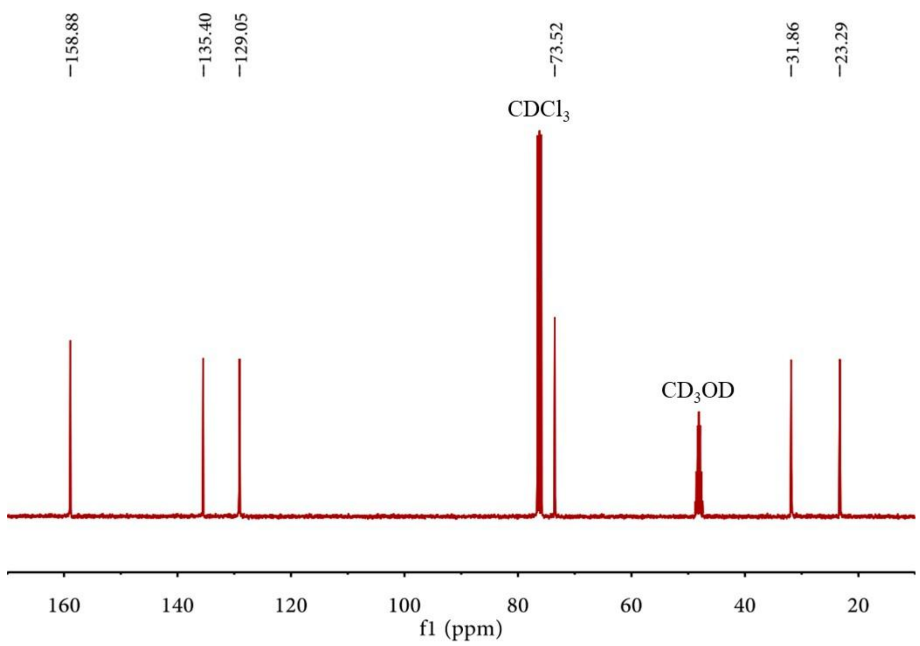

Supplementary Figure 5. ^{13}C-NMR spectrum of CC3 cage in $CD_3OD/CDCl_3$ (1:1) mixture solvent.

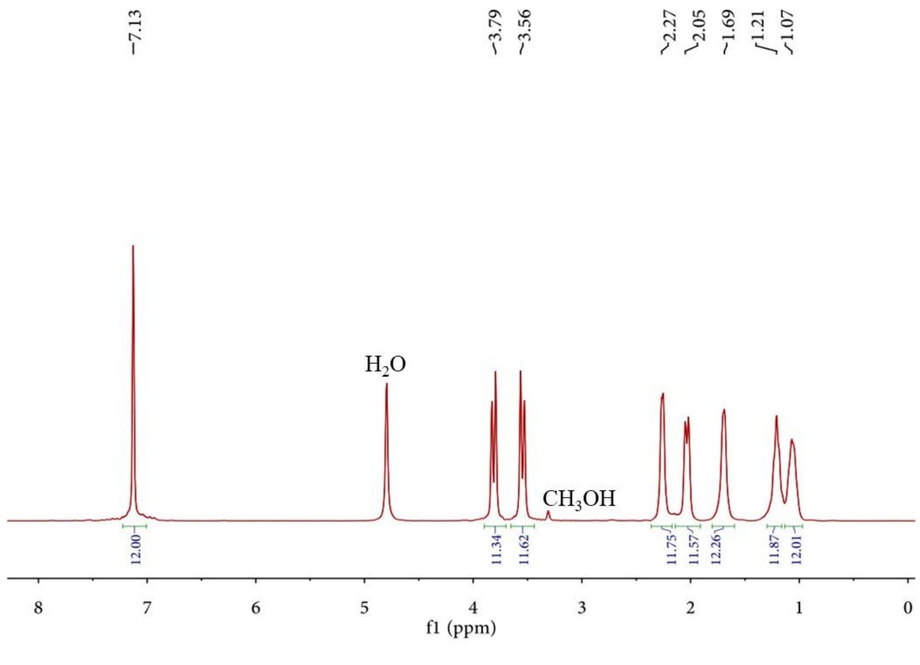

Supplementary Figure 6. ^{1}H-NMR spectrum of RCC3 cage in CD_3OD solvent.

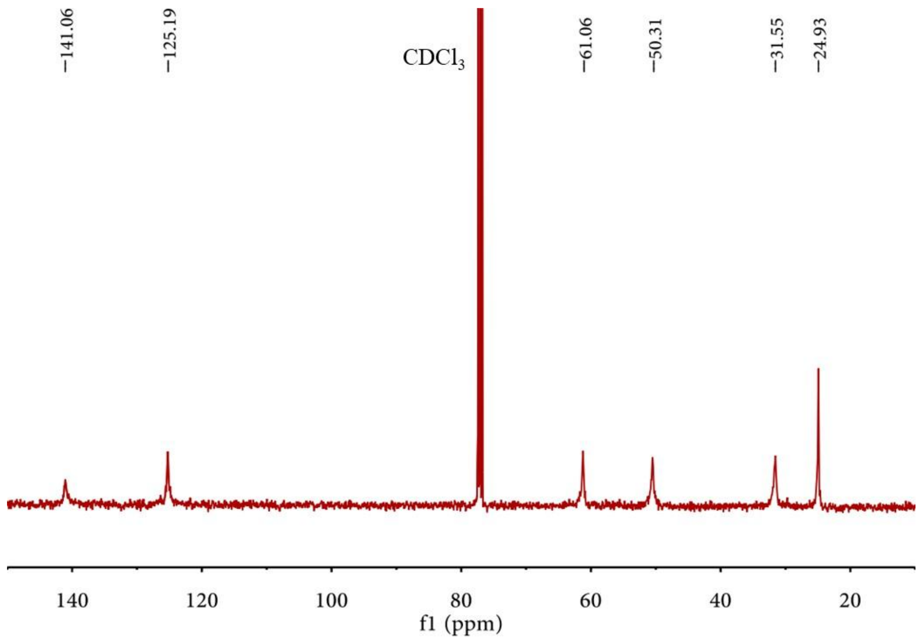

Supplementary Figure 7. ^{13}C-NMR spectrum of RCC3 cage in $CDCl_3$ solvent.

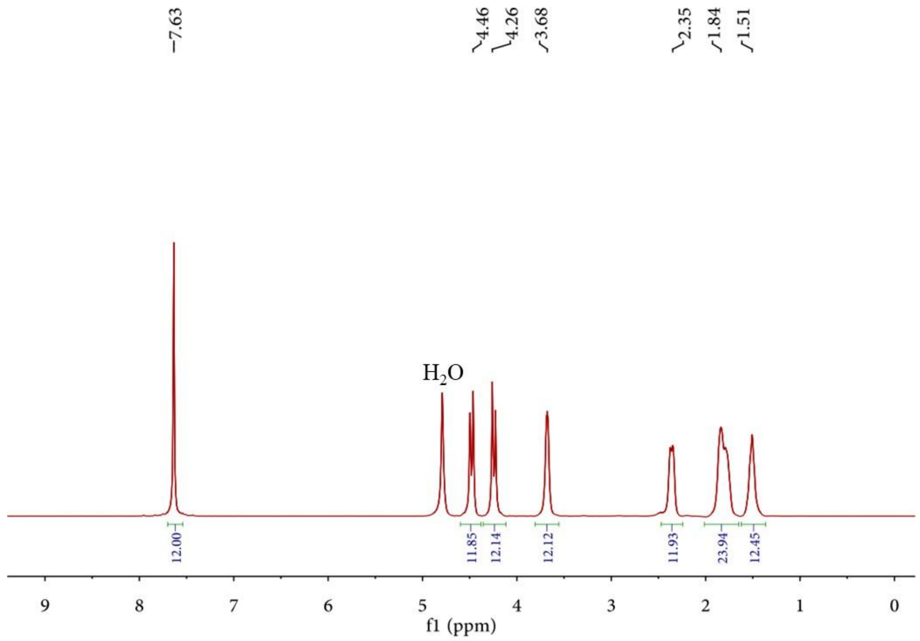

Supplementary Figure 8. ^{1}H-NMR spectrum of cationic C-Cage in D_2O solvent.

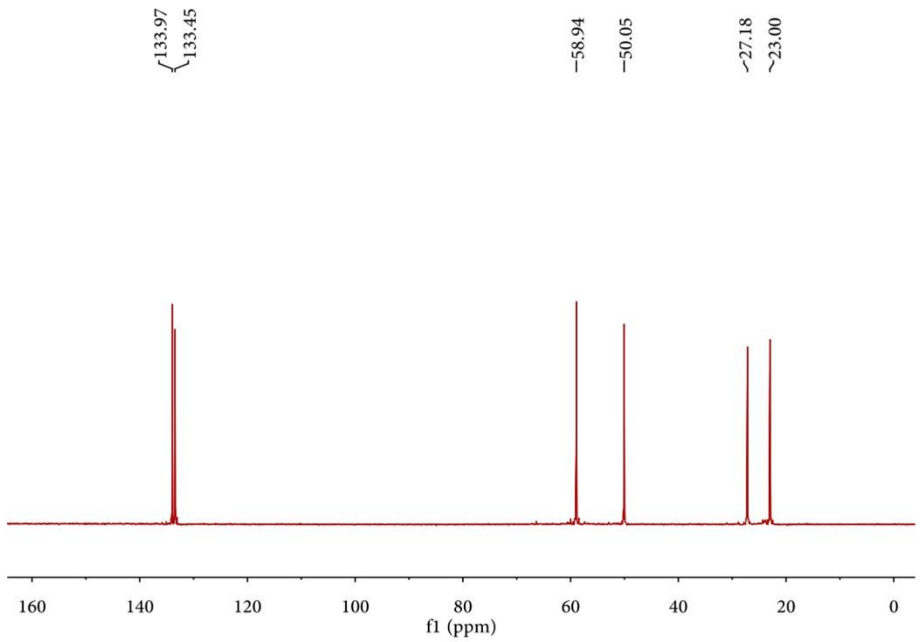

Supplementary Figure 9. ^{13}C-NMR spectrum of cationic C-Cage in D_2O solvent.

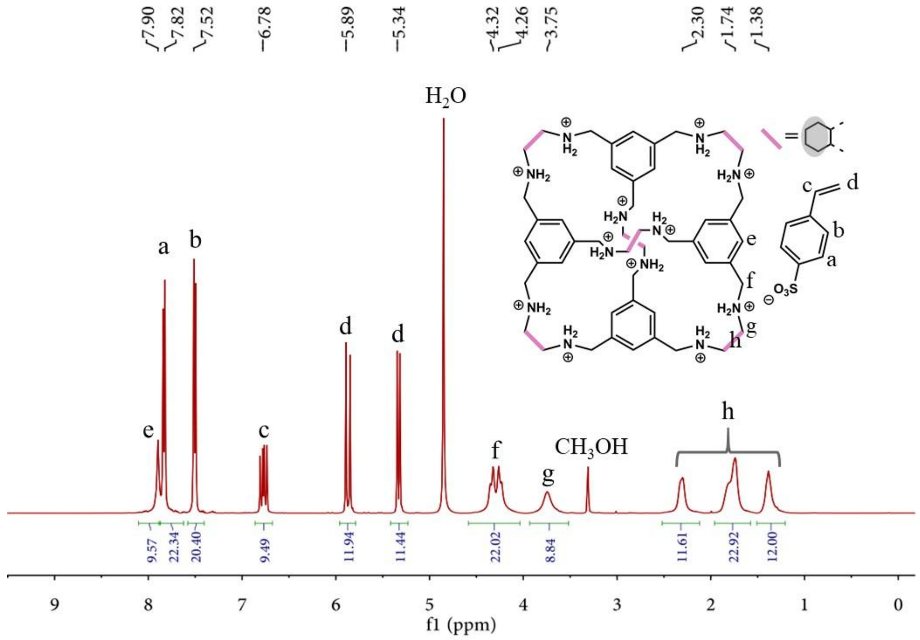

Supplementary Figure 10. ^{1}H-NMR spectrum of C-Cage-SS-12 in CD_3OD solvent (molar ratio of SS counteranion to cationic C-Cage is determined to be 12:1).

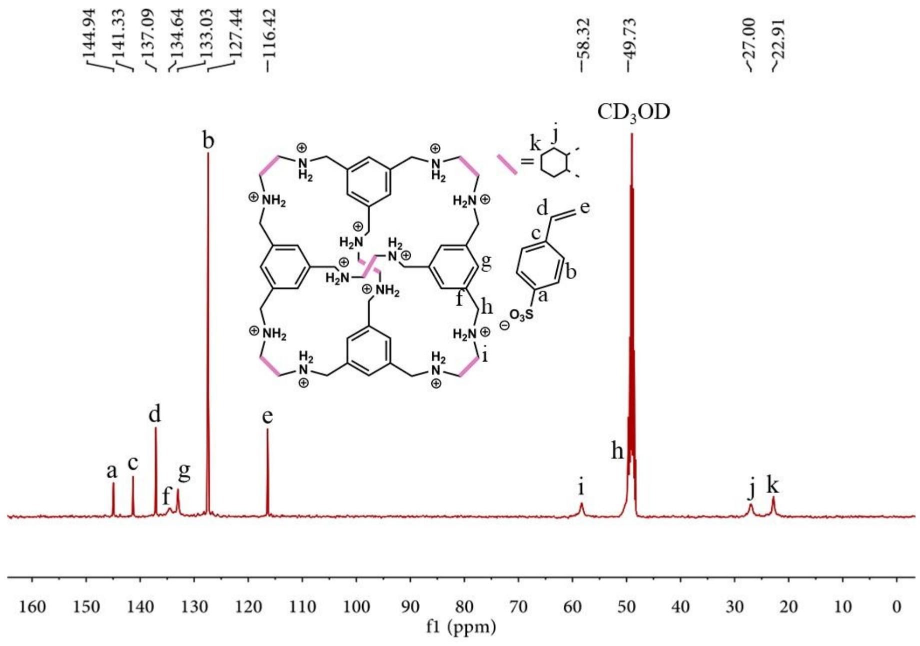

Supplementary Figure 11. ^{13}C-NMR spectrum of C-Cage-SS-12 in CD$_3$OD solvent.

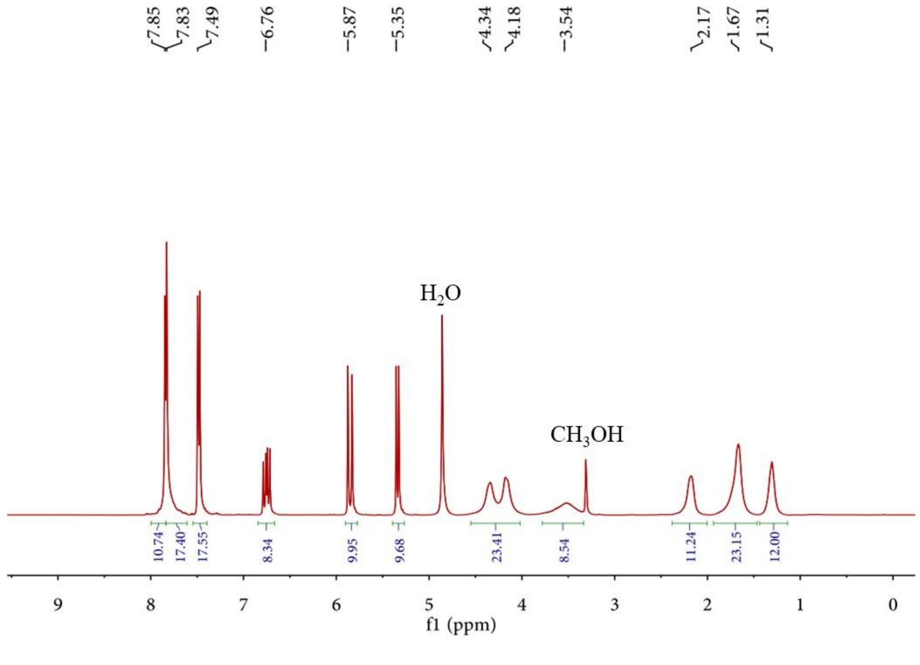

Supplementary Figure 12. ^{1}H-NMR spectrum of C-Cage-SS-9 in CD$_3$OD solvent (molar ratio of SS to C-Cage is determined to be 9:1).

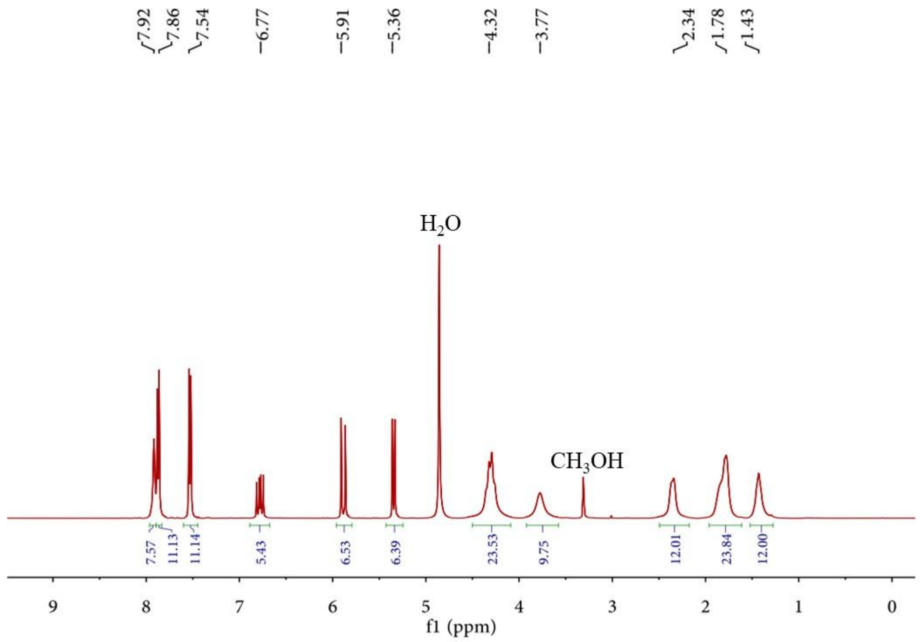

Supplementary Figure 13. ^{1}H-NMR spectrum of C-Cage-SS-6 in CD_3OD solvent (molar ratio of SS to C-Cage is determined to be 6:1).

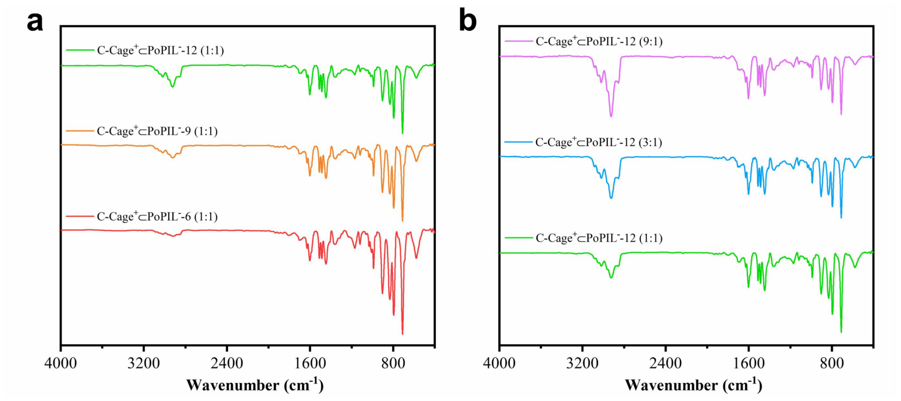

Supplementary Figure 14. FT-IR spectra of the C-Cage^{+}⊂PoPIL^{-} with (a) different ion exchange ratio (molar ratios of SS to C-Cage are 12:1, 9:1 and 6:1, in which the mass ratio of DVB crosslinker to C-Cage-SS is fixed at 1:1, the corresponding composite is denoted as C-Cage^{+}⊂PoPIL^{-}-Y, Y=12, 9 or 6) and (b) different DVB crosslinker amount (mass ratio of DVB to C-Cage-SS is 1:1, 3:1 and 9:1, in which the molar ratio of SS to C-Cage is fixed at 12:1).

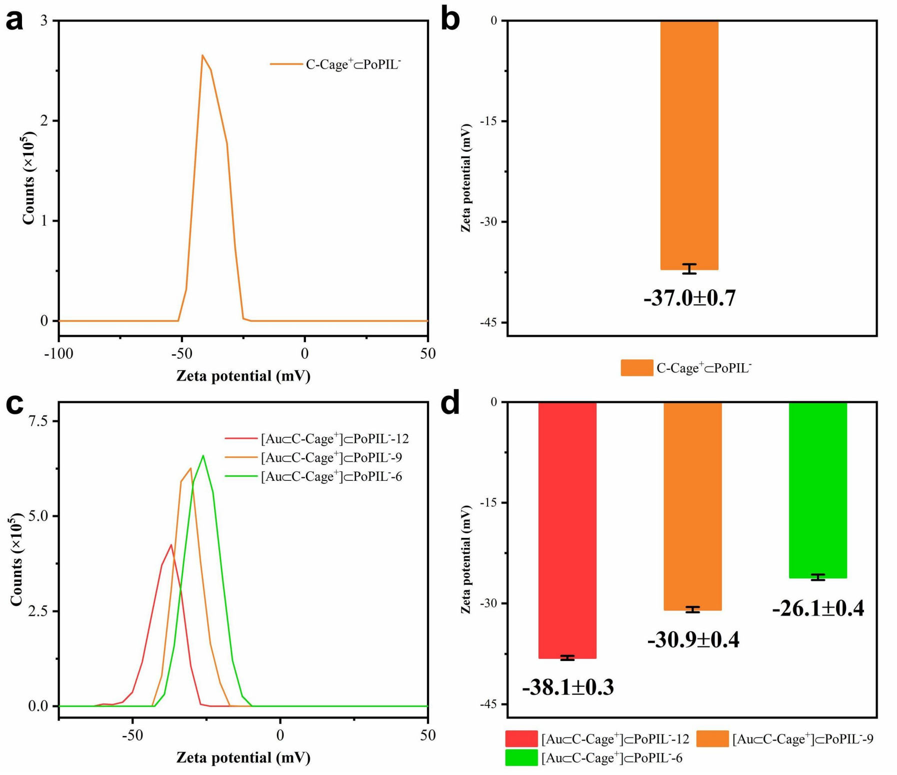

Supplementary Figure 15. Zeta potential curve (a) and the average value (b) of the C-Cage^{+}⊂PoPIL^{-} (molar ratio of SS to C-Cage is 12:1, mass ratio of DVB to C-Cage-SS is 1:1), zeta potential curve (c) and the average value (d) of the [Au⊂C-Cage^{+}]⊂PoPIL^{-}-Y (Y=12, 9 or 6, mass ratio of DVB to Au⊂C-Cage-SS is 1:1)

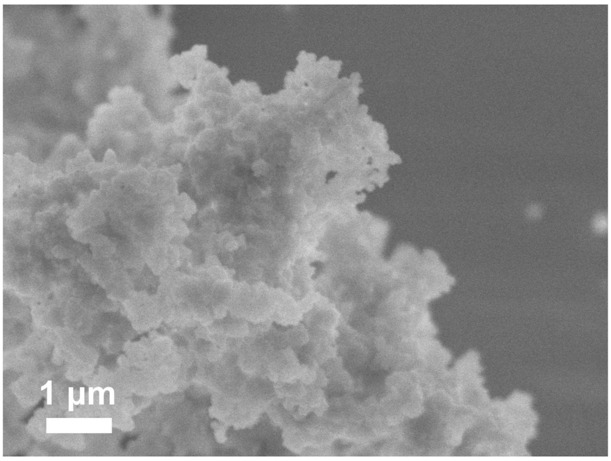

Supplementary Figure 16. SEM image of the C-Cage^{+}⊂PoPIL^{-} (molar ratio of SS to C-Cage is 12:1, mass ratio of DVB to C-Cage-SS is 1:1), scale bar: 1 μm.

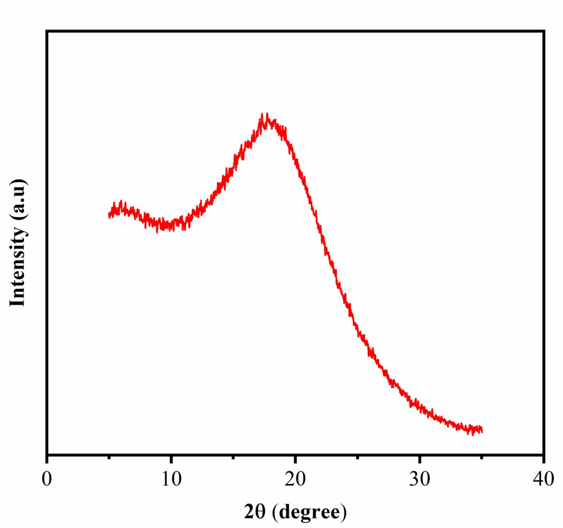

Supplementary Figure 17. PXRD pattern of the C-Cage$^+$⊂PoPIL$^-$ (molar ratio of SS to C-Cage is 12:1, mass ratio of DVB to C-Cage-SS is 1:1).

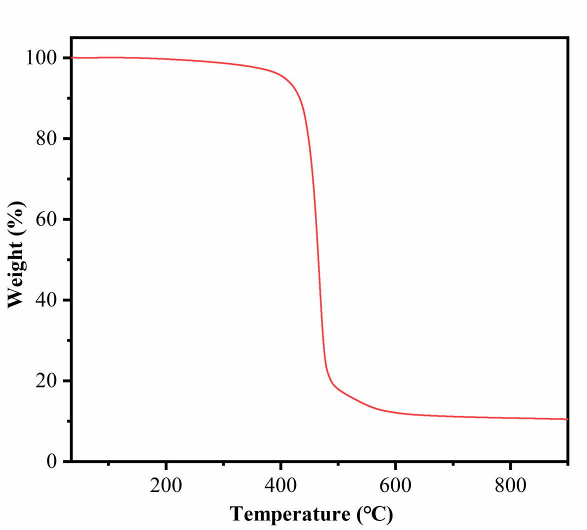

Supplementary Figure 18. TGA plot of the C-Cage$^+$⊂PoPIL$^-$ (molar ratio of SS to C-Cage is 12:1, mass ratio of DVB to C-Cage-SS is 1:1).

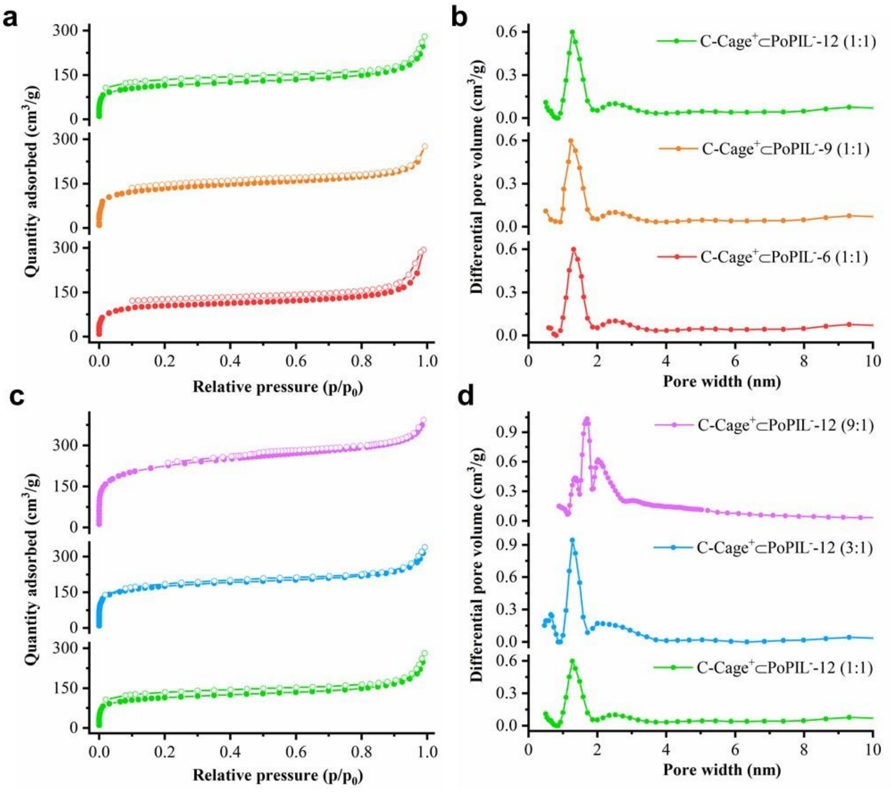

Supplementary Figure 19. N_2 sorption isotherms and NLDFT pore size distribution of (a, b) C-Cage$^+$⊂PoPIL$^-$-Y, Y=12, 9 or 6 (mass ratio of DVB to C-Cage-SS is fixed at 1:1) and (c, d) C-Cage$^+$⊂PoPIL$^-$ with variable DVB crosslinker amount (mass ratio of DVB to C-Cage-SS is 1:1, 3:1 and 9:1, in which the molar ratio SS to C-Cage is fixed at 12:1).

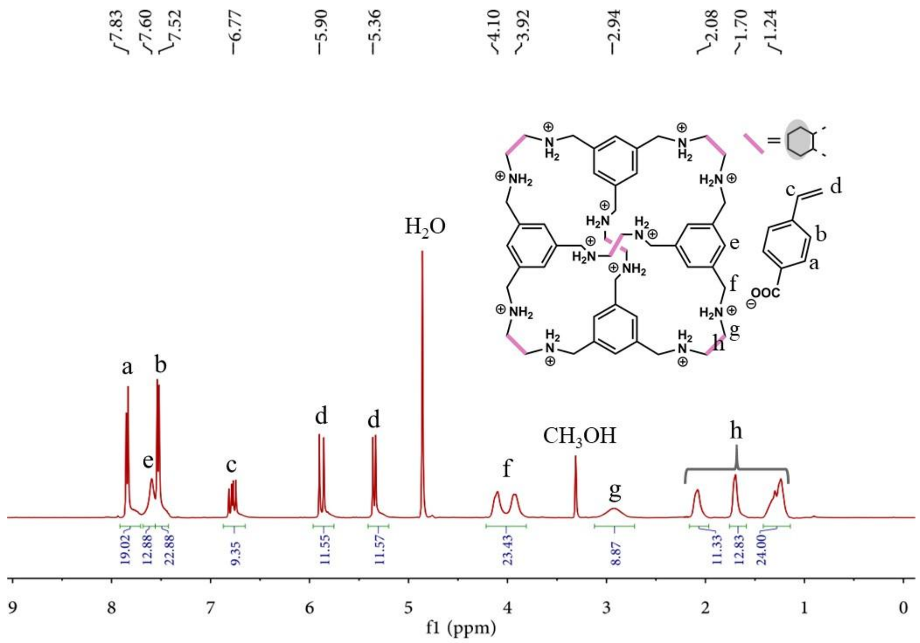

Supplementary Figure 20. ^{1}H-NMR spectrum of C-Cage-VB in CD_3OD solvent (molar ratio of VB to C-Cage is determined to be 12:1).

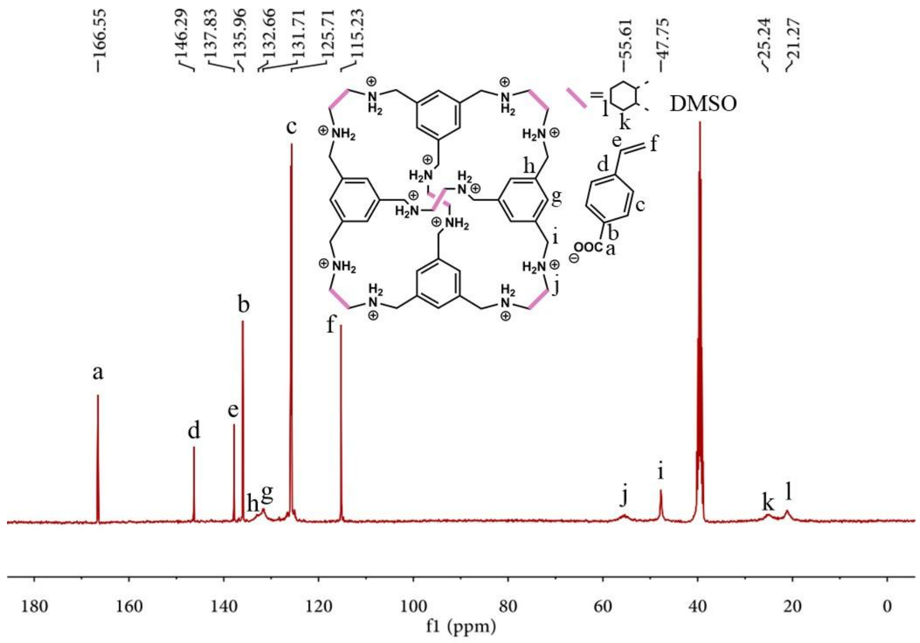

Supplementary Figure 21. ^{13}C-NMR spectrum of C-Cage-VB in d_6-DMSO solvent.

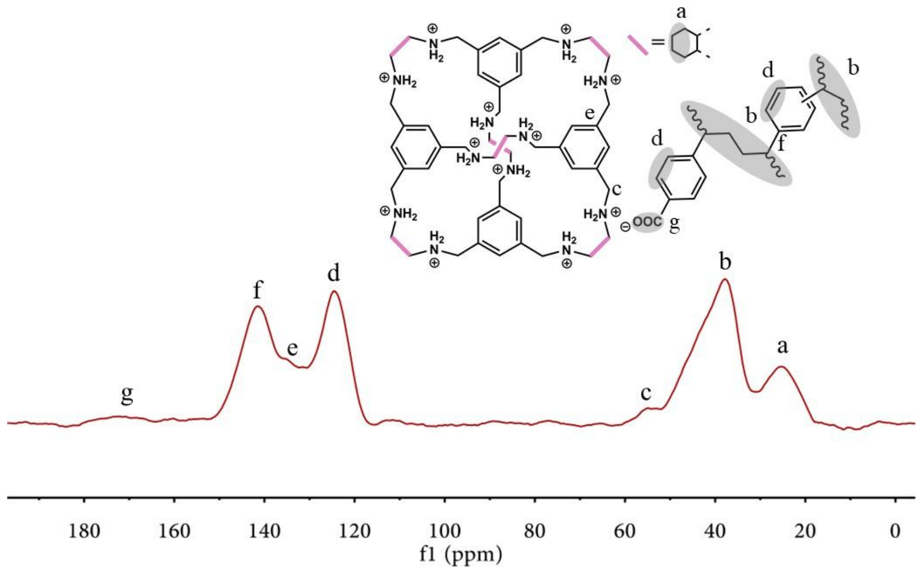

Supplementary Figure 22. ^{13}C CP/MAS solid-state NMR spectrum of C-Cage^{+}⊂PoPIL^{-} with VB monomer as ion exchange counteranion (molar ratio of VB to C-Cage is 12:1, mass ratio of DVB to C-Cage-VB is 1:1).

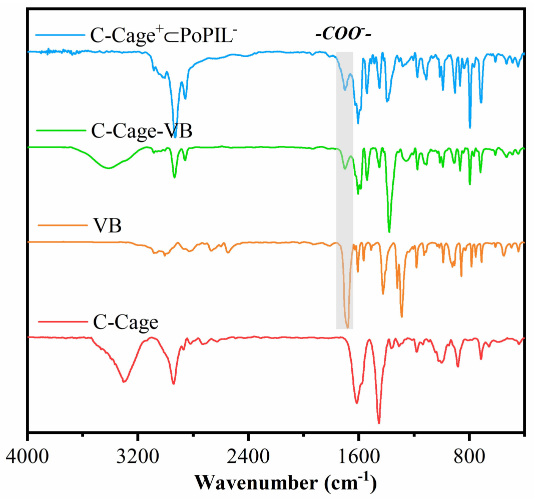

Supplementary Figure 23. FT-IR spectra of the C-Cage, VB, C-Cage-VB and C-Cage$^+$⊂PoPIL$^-$ with VB monomer as ion exchange counteranion (molar ratio of VB to C-Cage is 12:1, mass ratio of DVB to C-Cage-VB is 1:1).

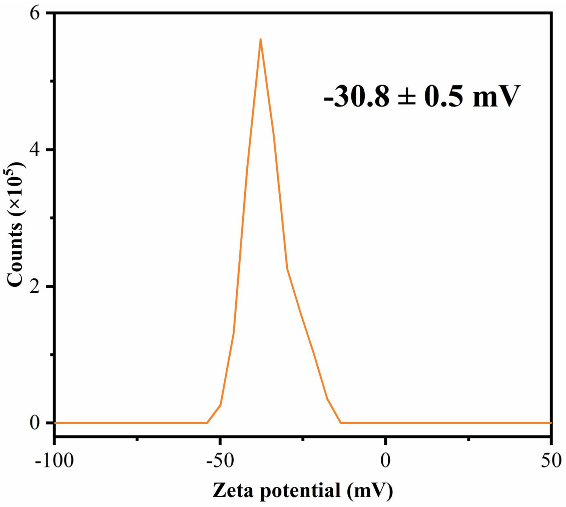

Supplementary Figure 24. Zeta potential curve of the C-Cage$^+$⊂PoPIL with VB monomer as ion exchange counteranion (molar ratio of VB to C-Cage is 12:1, mass ratio of DVB to C-Cage-VB is 1:1).

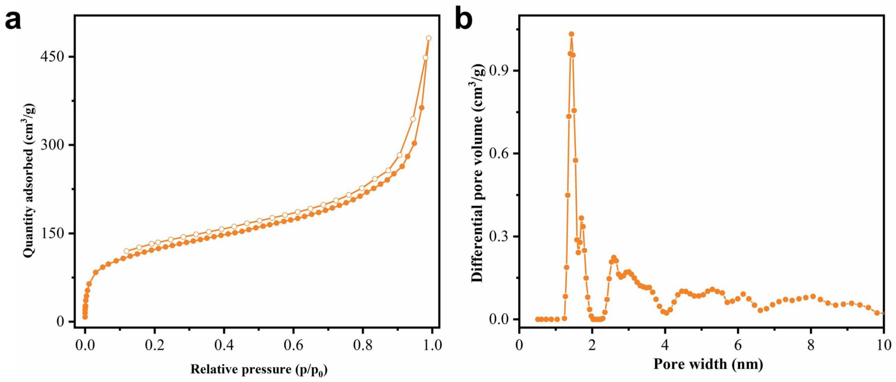

Supplementary Figure 25. (a) N_2 sorption isotherms and (b) NLDFT pore size distribution plot of the C-Cage$^+$⊂PoPIL$^-$ with VB monomer as ion exchange counteranion (molar ratio of VB to C-Cage is 12:1, mass ratio of DVB to C-Cage-VB is 1:1).

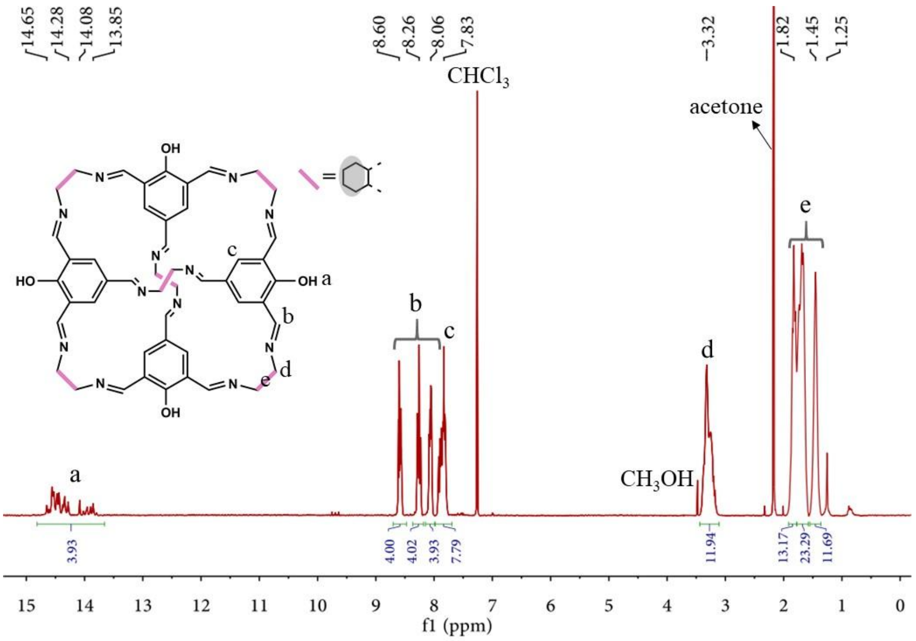

Supplementary Figure 26. ^{1}H-NMR spectrum of CC3-OH cage in $CD_3OD/CDCl_3$ (1:1) mixture solvent.

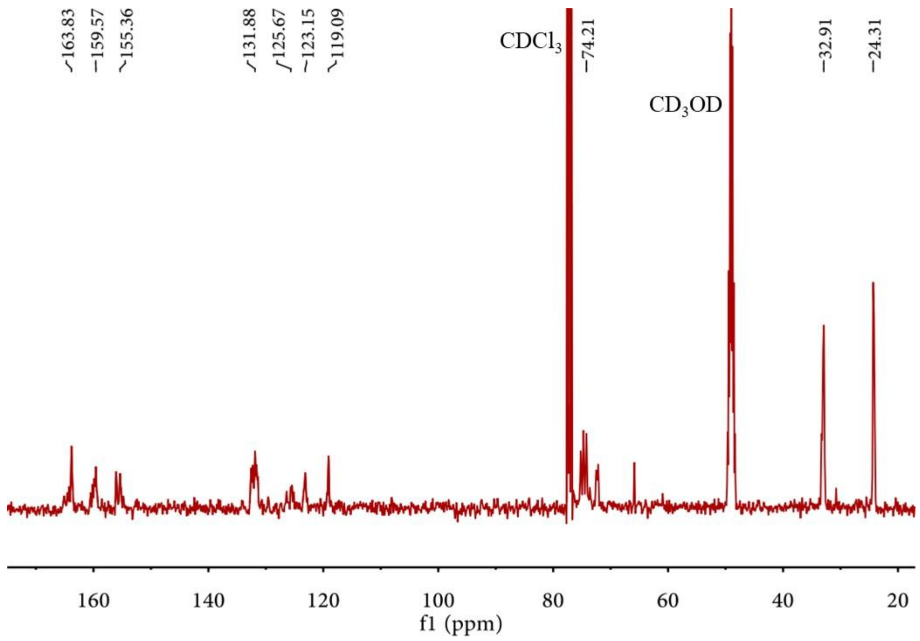

Supplementary Figure 27. ^{13}C-NMR spectrum of CC3-OH cage in $CD_3OD/CDCl_3$ (1:1) mixture solvent.

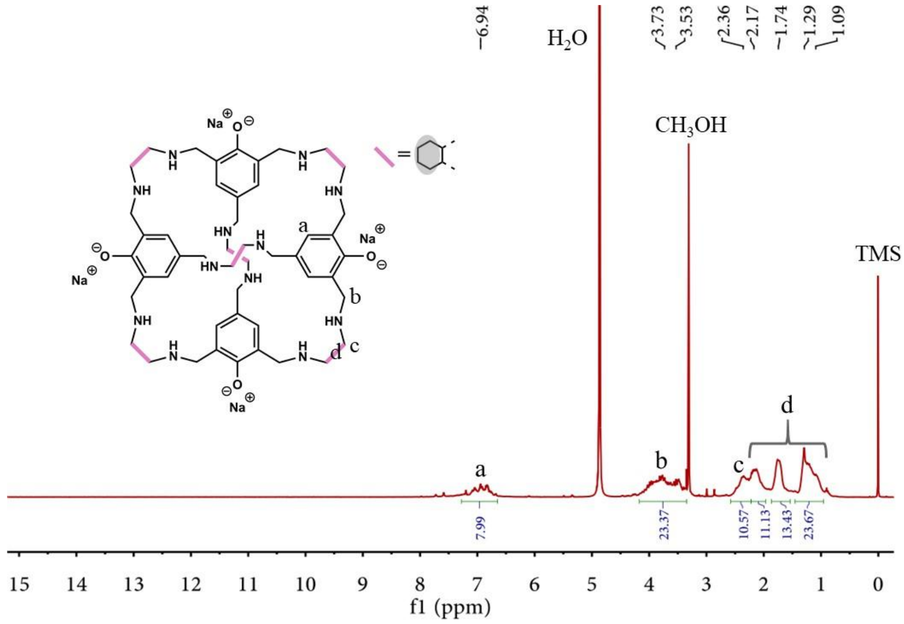

Supplementary Figure 28. ^{1}H-NMR spectrum of anionic A-Cage in CD_3OD solvent.

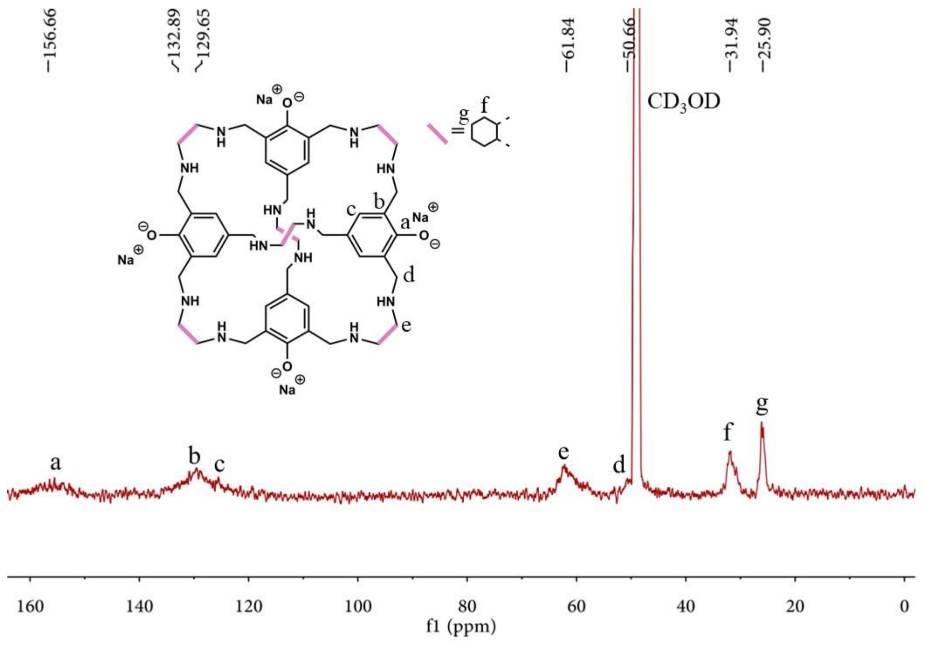

Supplementary Figure 29. ^{13}C-NMR spectrum of anionic A-Cage in CD_3OD solvent.

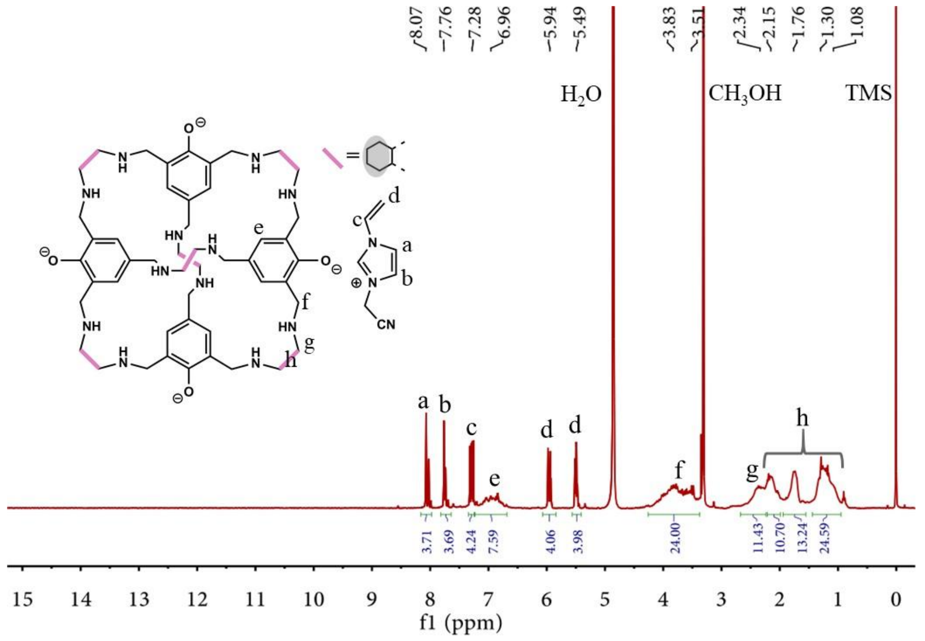

Supplementary Figure 30. ^{1}H-NMR spectrum of A-Cage-imidaz in CD_3OD solvent (molar ratio of imidaz to A-Cage is determined to be 4:1).

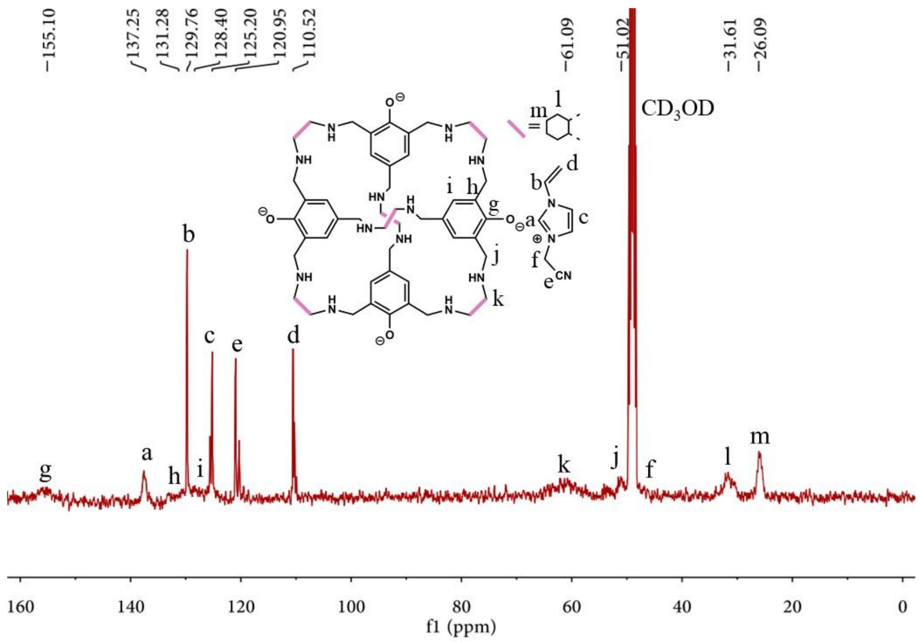

Supplementary Figure 31. ^{13}C-NMR spectrum of A-Cage-imidaz in CD_3OD solvent.

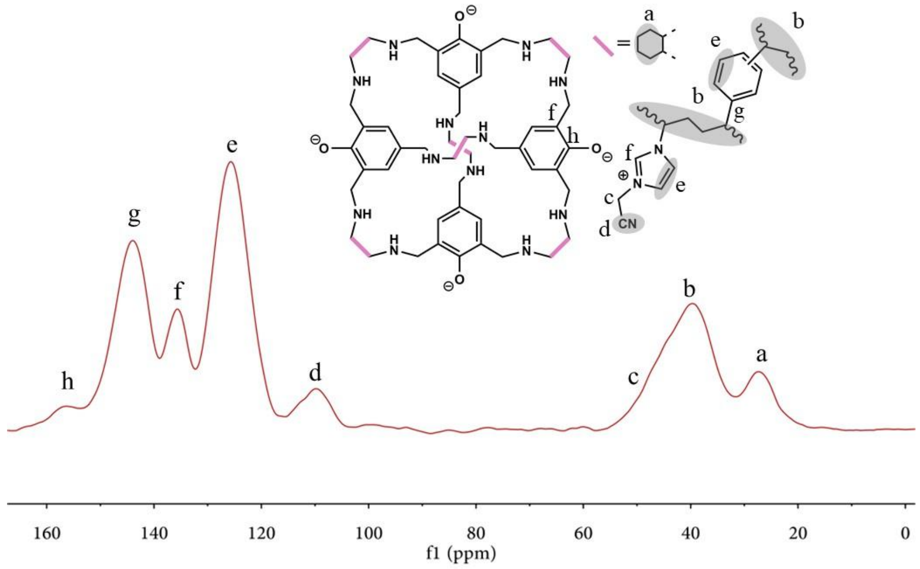

Supplementary Figure 32. ^{13}C CP/MAS solid-state NMR spectrum of A-Cage^{-}⊂PoPIL^{+} with imidaz monomer as ion exchange countercation (molar ratio of imidaz to A-Cage is 4:1, mass ratio of DVB to A-Cage-imidaz is 1:1).

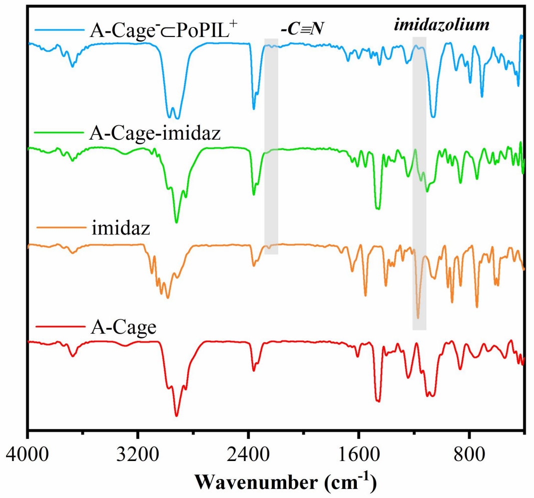

Supplementary Figure 33. FT-IR spectra of the A-Cage, imidaz, A-Cage-imidaz and A-Cage$^-$⊂PoPIL$^+$ (molar ratio of imidaz to A-Cage is 4:1, mass ratio of DVB to A-Cage-imidaz is 1:1).

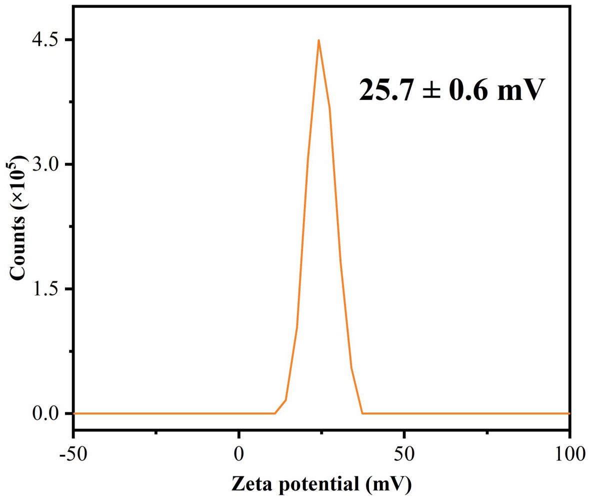

Supplementary Figure 34. Zeta potential curve of the A-Cage$^-$⊂PoPIL$^+$ (molar ratio of imidaz to A-Cage is 4:1, mass ratio of DVB to A-Cage-imidaz is 1:1).

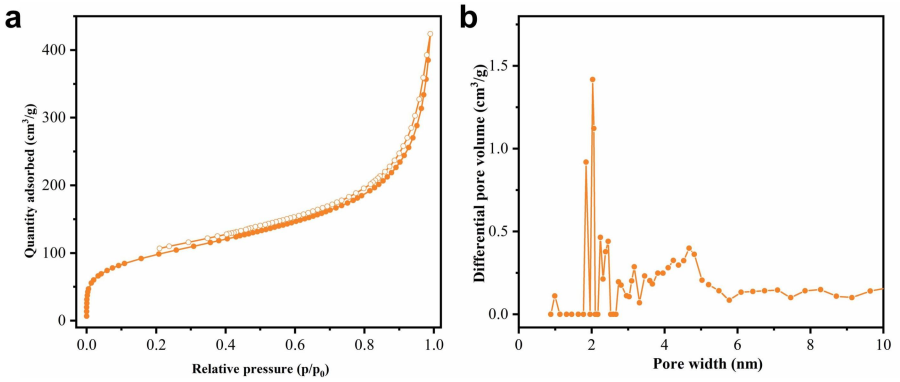

Supplementary Figure 35. (a) N_2 sorption isotherms and (b) NLDFT pore size distribution plot of the A-Cage$^-$⊂PoPIL$^+$ (molar ratio of imidaz to A-Cage is 4:1, mass ratio of DVB to A-Cage-imidaz is 1:1).

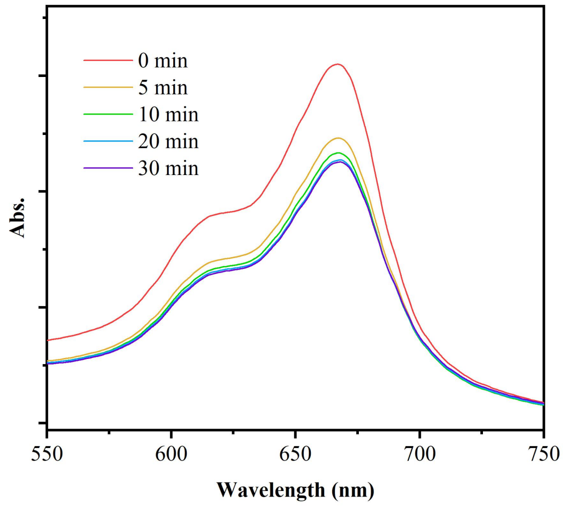

Supplementary Figure 36. UV-Vis spectra of MB substrate solution after adsorption by [Au⊂C-Cage$^+$]⊂PoPIL$^-$. The characteristic peak of MB at 665 nm decreased its intensity from 0 to 20 min, which remained constant after 20 min, indicating an approximate equilibrium of adsorption has been reached.

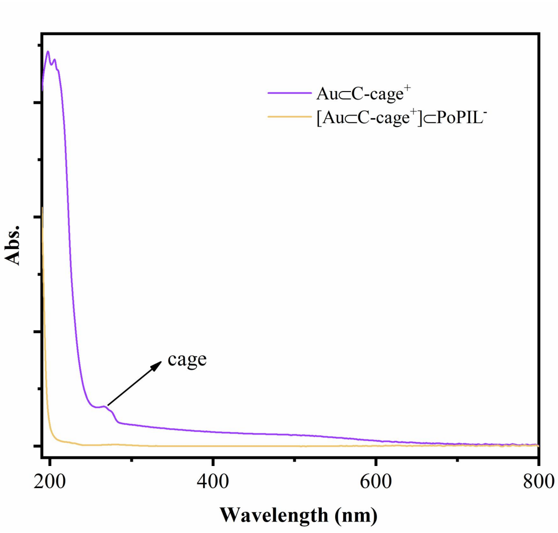

Supplementary Figure 37. UV-Vis spectra of Au⊂C-Cage$^+$ and [Au⊂C-Cage$^+$]⊂PoPIL$^-$ in water. The characteristic peak of ionic cage molecule at 295 nm is not observed for [Au⊂C-Cage$^+$]⊂PoPIL$^-$ catalyst after dispersed in water over 24 h, demonstrating the chemical stability of the Au⊂C-Cage$^+$ inner host after entrapped inside the PoPIL$^-$.

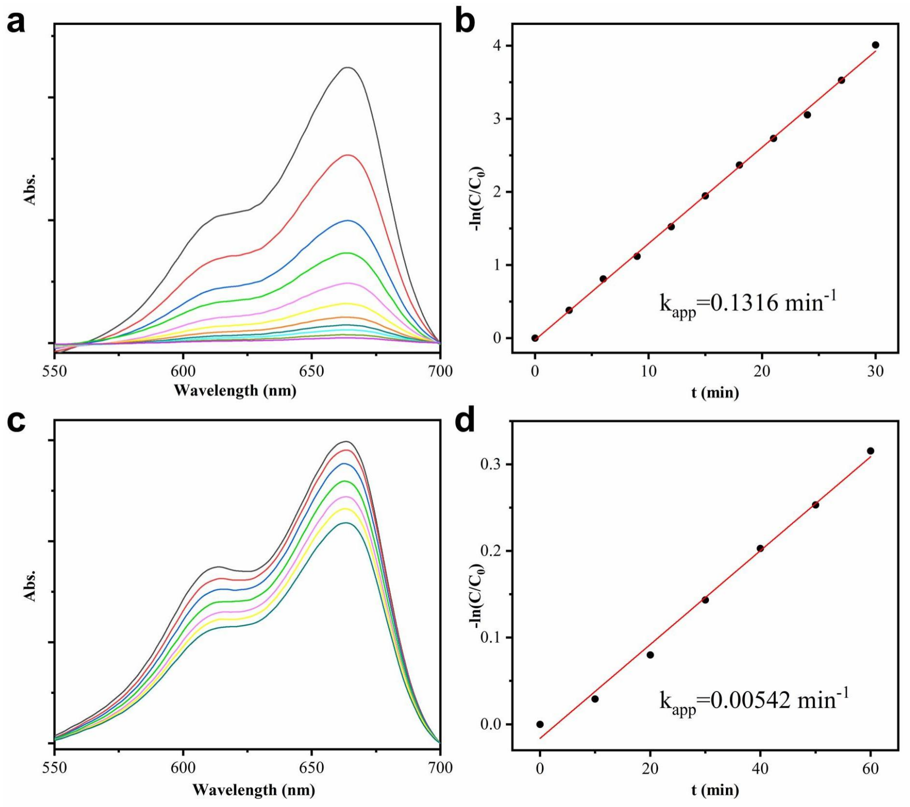

Supplementary Figure 38. (a) UV-Vis spectra and (b) linear fitting of the reaction rate constant for degradation of positively charged MB by [Au⊂C-Cage$^+$]⊂PoPIL$^-$ catalyst, (c) UV-Vis spectra and (d) linear fitting of reaction rate constant for MB degradation by nonconfined Au⊂C-Cage$^+$ catalyst.

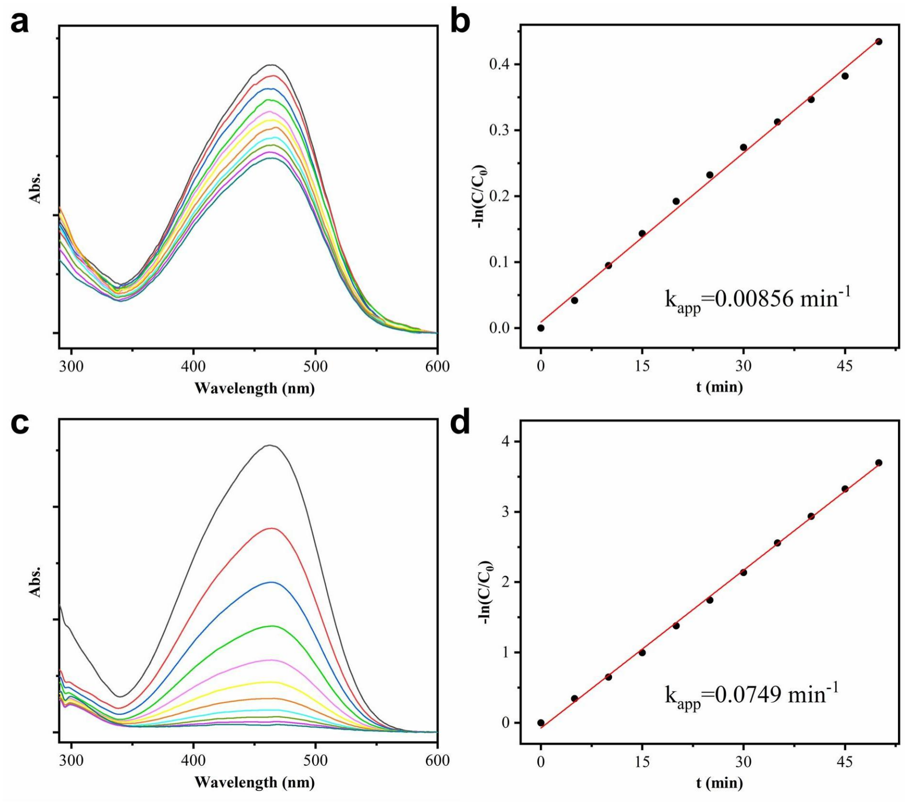

Supplementary Figure 39. (a) UV-Vis spectra and (b) linear fitting of the reaction rate constant for degradation of negatively charged MO by [Au⊂C-Cage$^+$]⊂PoPIL$^-$ catalyst, (c) UV-Vis spectra and (d) linear fitting of reaction rate constant for MO degradation by nonconfined Au⊂C-Cage$^+$ catalyst.

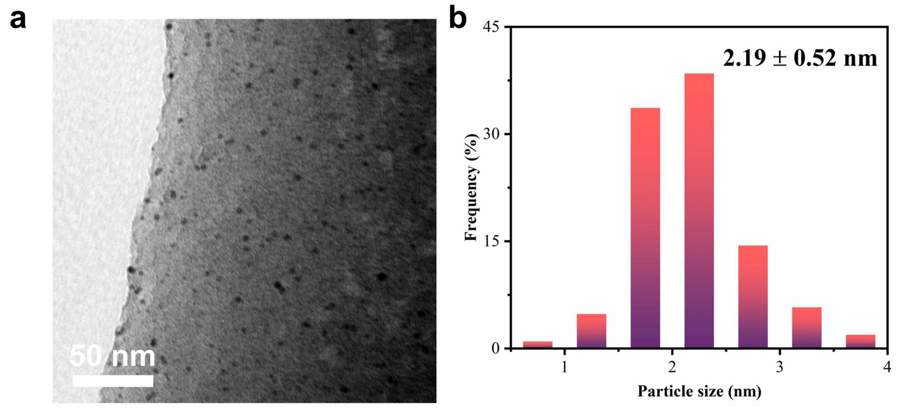

Supplementary Figure 40. (a) TEM image (scale bar: 50 nm) and (b) statistic particle size distribution histogram of Au nanoparticles in Au⊂PoPIL$^-$ (data calculated from 104 counts).

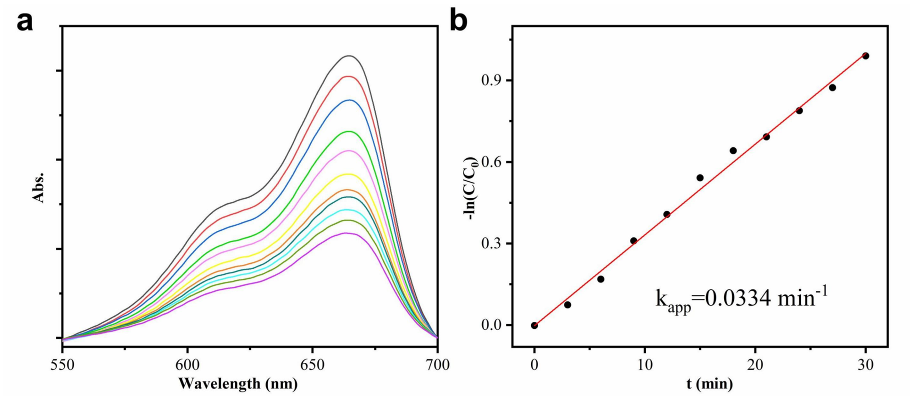

Supplementary Figure 41. (a) UV-Vis spectra and (b) linear fitting of reaction rate constant for degradation of positively charged MB by Au⊂PoPIL⁻ catalyst. The Au content in Au⊂PoPIL⁻ catalyst was the same as that of [Au⊂C-Cage^{+}]⊂PoPIL⁻, the exact mass of 3.3 mg was calculated from ICP results (Supplementary Table 4).

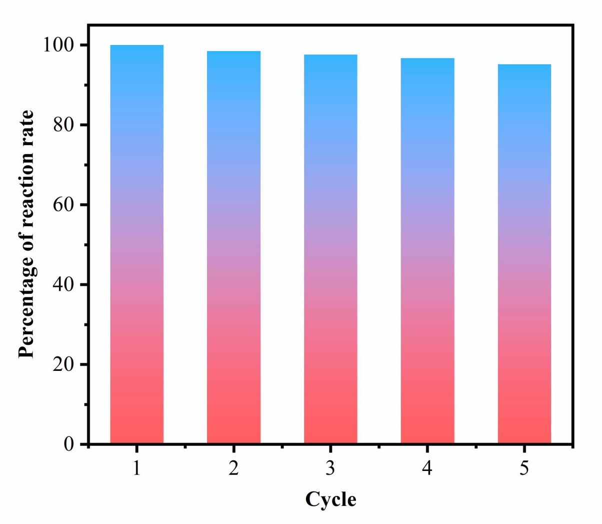

Supplementary Figure 42. Catalytic activities of [Au⊂C-Cage^{+}]⊂PoPIL⁻ catalyst for 5 successive cycles in the MB degradation reaction.

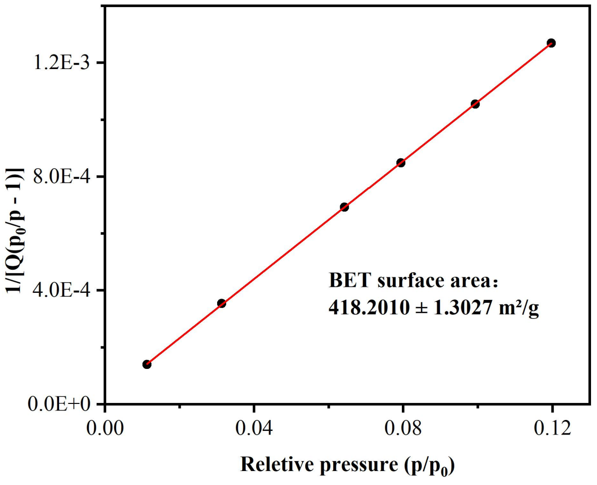

Supplementary Figure 43. Determination of the specific surface area (according to the BET method) of [Au⊂C-Cage^{+}]⊂PoPIL^{-} after 5 cycles' catalytic reaction.

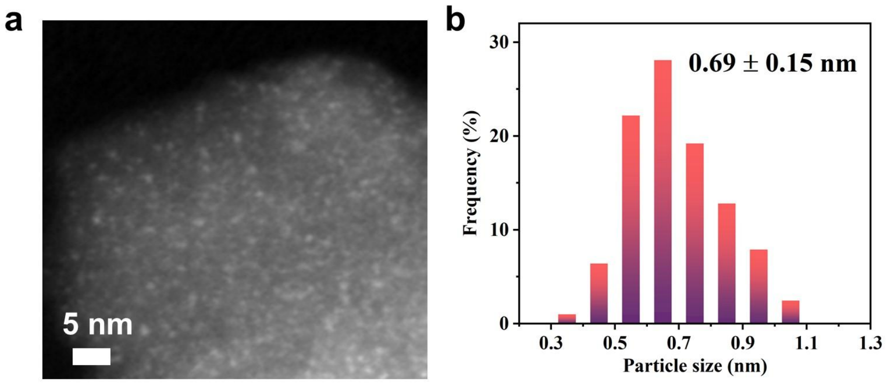

Supplementary Figure 44. (a) HAADF-STEM image of the Au clusters uniformly dispersed in the recycled [Au⊂C-Cage^{+}]⊂PoPIL^{-} catalyst, scale bar: 5 nm, (b) statistic size distribution histogram of Au clusters (data calculated from 203 counts).

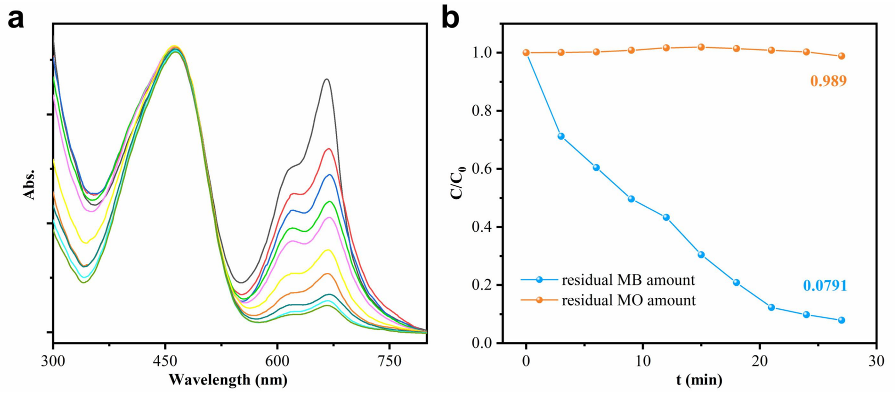

Supplementary Figure 45. (a) UV-Vis spectra of competitive decomposition of positive MB and negative MO substrates with an equal molar amount in solution, (b) residual substrate amount in the solution during the degradation reaction.

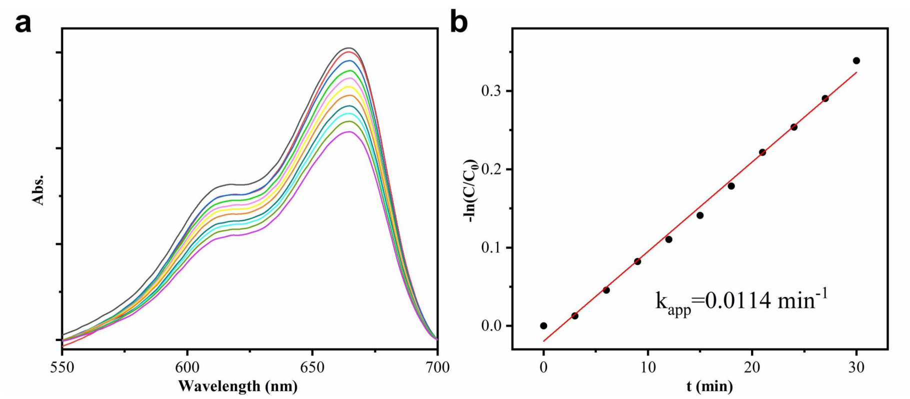

Supplementary Figure 46. (a) UV-Vis spectra and (b) linear fitting of reaction rate constant for degradation of positively charged MB by [Au⊂C-Cage^{+}]⊂PoPIL^{-} catalyst in 1X PBS solution.

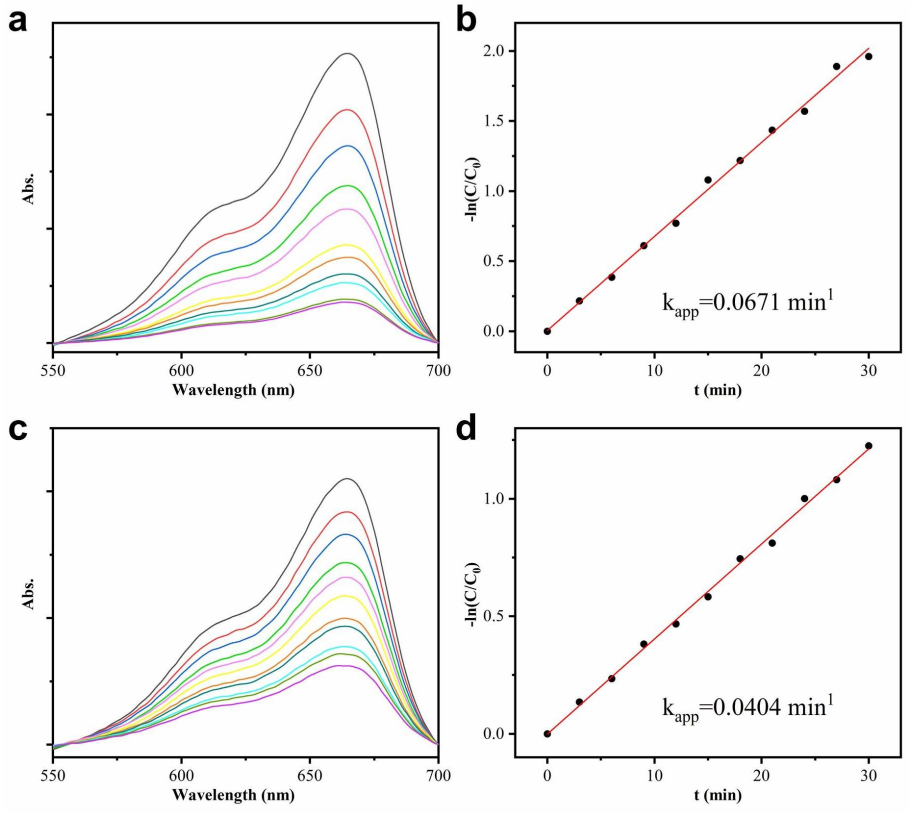

Supplementary Figure 47. (a) UV-Vis spectra and (b) linear fitting of reaction rate constant for degradation of positively charged MB by [Au⊂C-Cage^{+}]⊂PoPIL^{-}-9 (molar ratio of SS to Au⊂C-Cage^{+} is 9:1 in the anion exchange step prior to polymerization), (c) UV-Vis spectra and (d) linear fitting of reaction rate constant for MB degradation by [Au⊂C-Cage^{+}]⊂PoPIL^{-}-6 (molar ratio of SS to Au⊂C-Cage^{+} is 6:1 in the anion exchange step prior to polymerization).

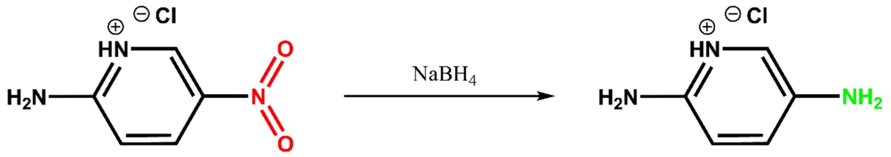

Supplementary Figure 48. Schematic illustration of the reduction of the cationic organic micropollutant 2A5NPCl by $NaBH_4$.

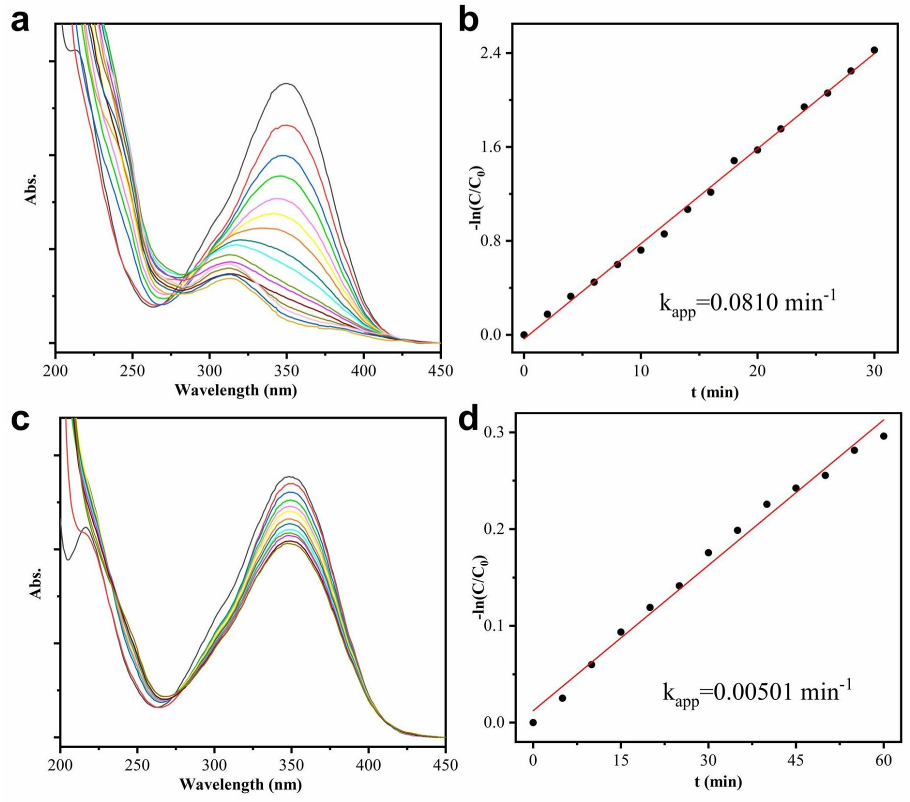

Supplementary Figure 49. (a) UV-Vis spectra and (b) linear fitting of reaction rate constant for positively charged 2A5NPCl reduced by [Au⊂C-Cage$^+$]⊂PoPIL$^-$ catalyst, (c) UV-Vis spectra and (d) linear fitting of reaction rate constant for 2A5NPCl reduced by nonconfined Au⊂C-Cage$^+$ catalyst.

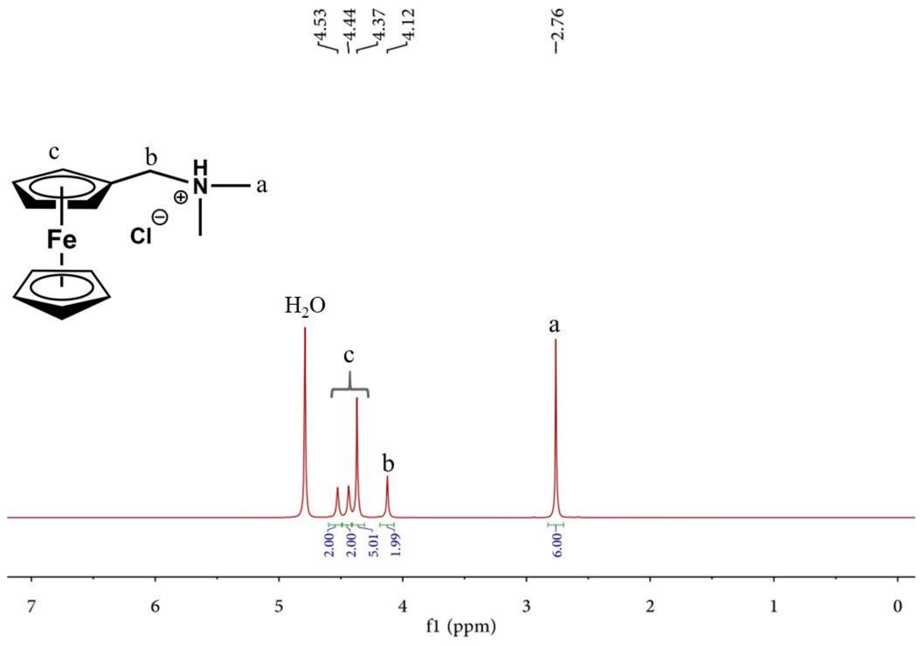

Supplementary Figure 50. ^{1}H-NMR spectrum of Fer^{+} in D_2O solvent.

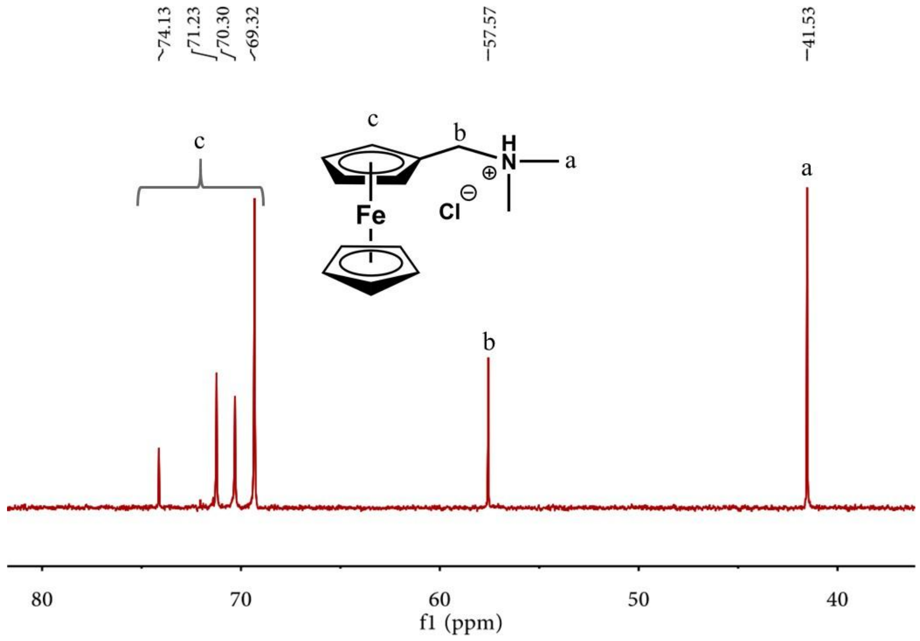

Supplementary Figure 51. ^{13}C-NMR spectrum of Fer^{+} in D_2O solvent.

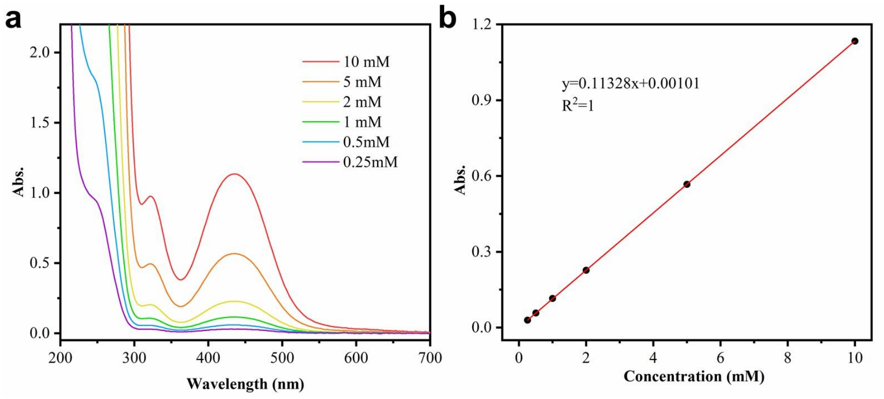

Supplementary Figure 52. (a) UV-Vis spectra of Fer^+ solution with different concentration, (b) linear fitting of working curve for Fer^+.

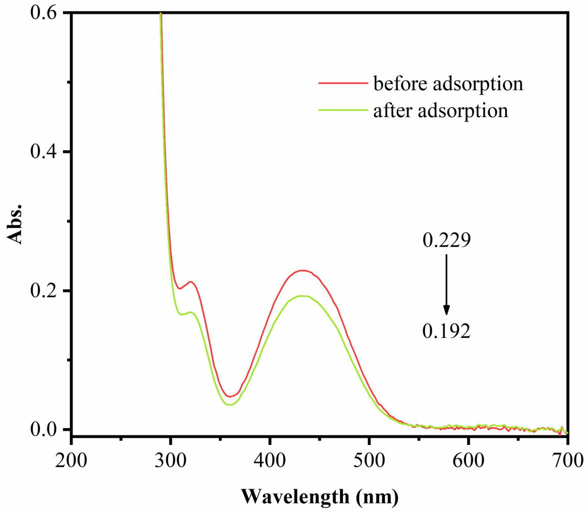

Supplementary Figure 53. UV-Vis spectra of Fer^+ solution before and after adsorption by [Au⊂C-Cage$^+$]⊂PoPIL$^-$, the calculated molar ratio of Fe to Au is ~2.15 according to the adsorption result which is in consistent with the ICP-OES results (Supplementary Table 4).

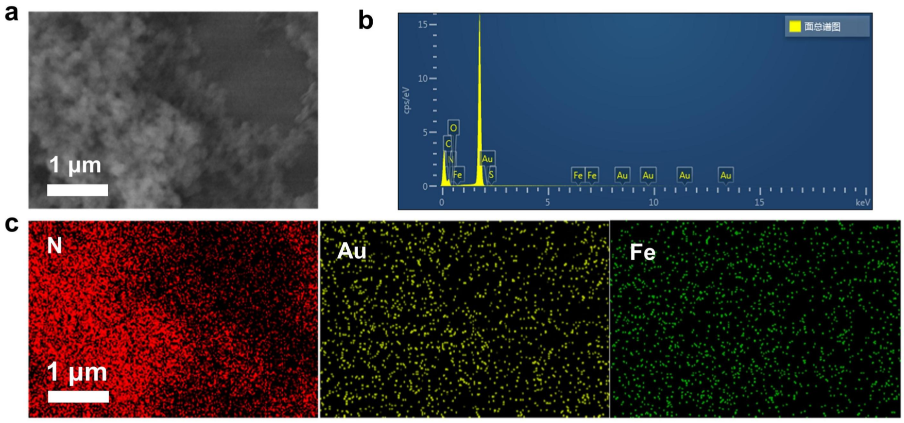

Supplementary Figure 54. (a) SEM image of the [Au⊂C-Cage^{+}&Fer^{+}]⊂PoPIL^{-}, scale bar: 1 μm, (b) EDS elemental analysis results, (c) elemental mapping of N, Au and Fe elements, scale bar: 1 μm.

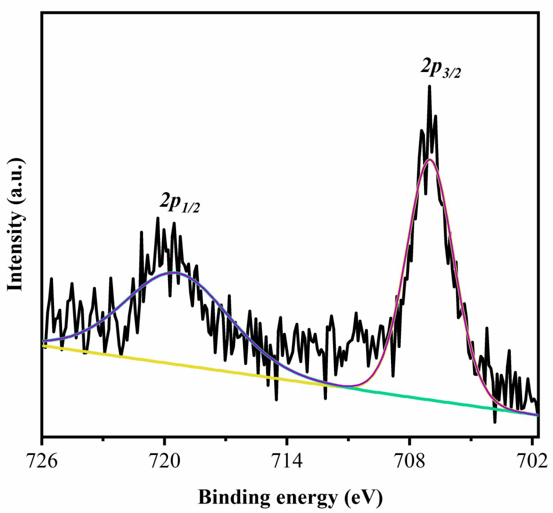

Supplementary Figure 55. XPS spectrum showing the Fe $2p_{1/2}$ and $2p_{3/2}$ peaks in [Au⊂C-Cage^{+}&Fer^{+}]⊂PoPIL^{-} catalyst.

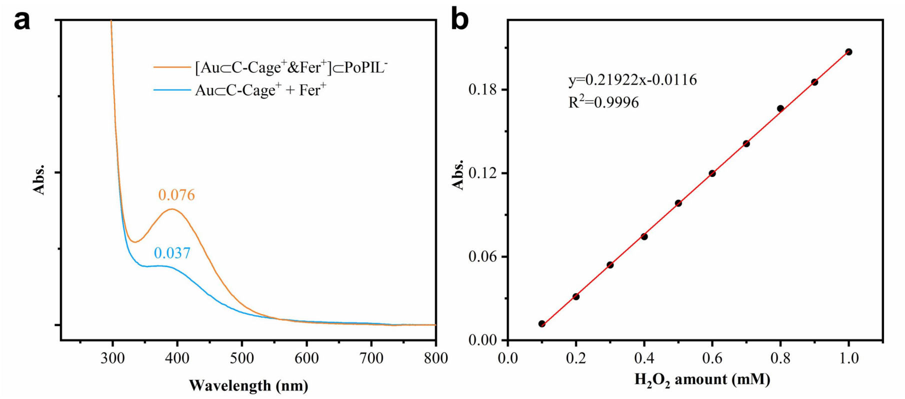

Supplementary Figure 56. (a) UV-Vis spectra of H_2O_2 generated by confined [Au⊂C-Cage$^+$&Fer$^+$]⊂PoPIL$^-$ catalyst and a mixture of free Au⊂C-Cage$^+$ and Fer$^+$ catalysts in the solution, (b) linear fitting of working curve for H_2O_2.

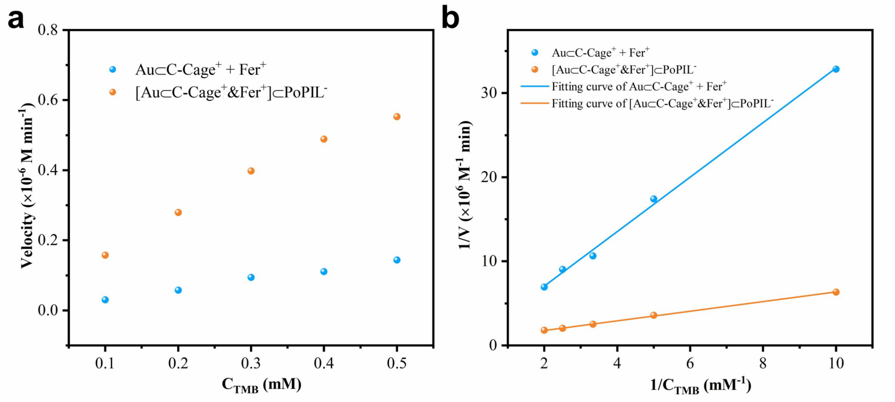

Supplementary Figure 57. (a) Comparison of initial reaction velocity and (b) linear fitting of double reciprocal plots for confined [Au⊂C-Cage$^+$&Fer$^+$]⊂PoPIL$^-$ catalyst and a mixture of free Au⊂C-Cage$^+$ and Fer$^+$ catalysts against different TMB concentration.

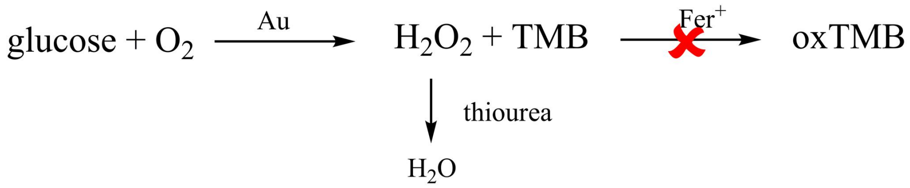

Supplementary Figure 58. Schematic illustration for the investigation of the substrate channelling effect by adding a competing reagent (thiourea) that can consume the H_2O_2 intermediate.

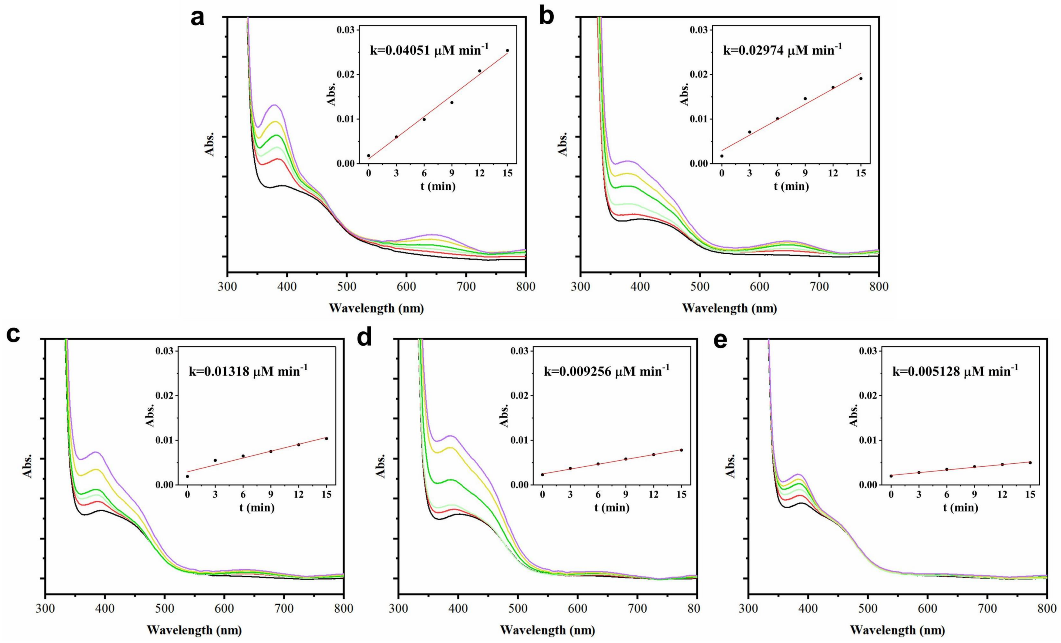

Supplementary Figure 59. UV-Vis spectra and linear fitting of reaction rate constant (inserted figures) of the enzymatic-like cascade TMB oxidation reaction with the addition of thiourea as competing reagent by a mixture of free Au⊂C-Cage^{+} and Fer^{+} catalysts in the solution. Concentrations of the competing reagent are (a) 0/blank, (b) 0.5 mM, (c) 1 mM, (d) 1.5 mM and (e) 2 mM respectively.

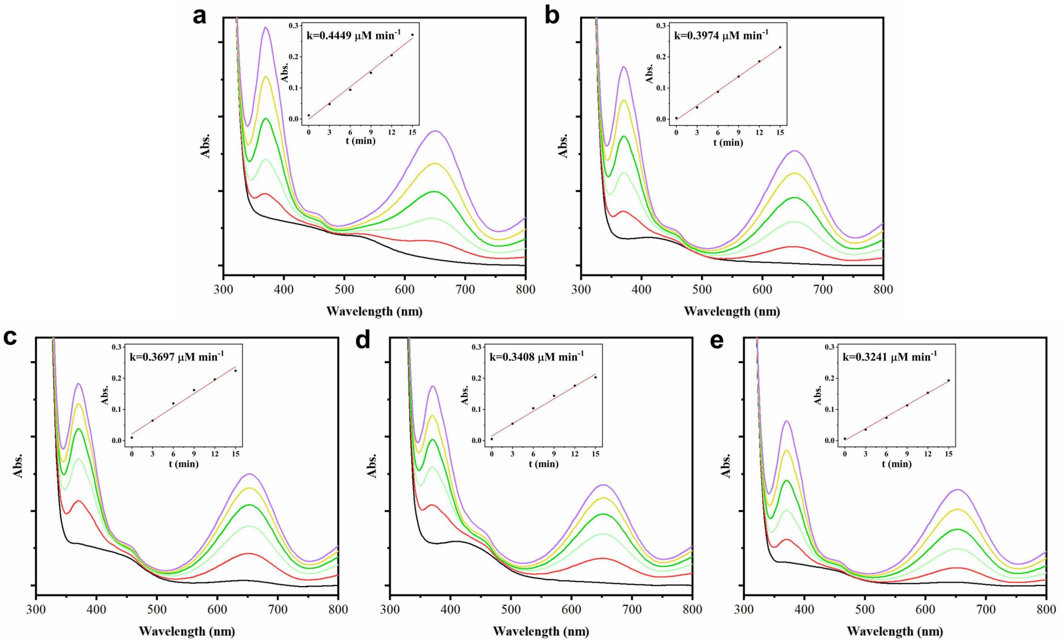

Supplementary Figure 60. UV-Vis spectra and linear fitting of reaction rate constant (inserted figures) of the enzymatic-like cascade TMB oxidation reaction with the addition of thiourea as competing reagent by confined [Au⊂C-Cage^{+}&Fer^{+}]⊂PoPIL^{-} catalyst. Concentrations of the competitor reagent are (a) 0/blank, (b) 0.5 mM, (c) 1 mM, (d) 1.5 mM and (e) 2 mM respectively.

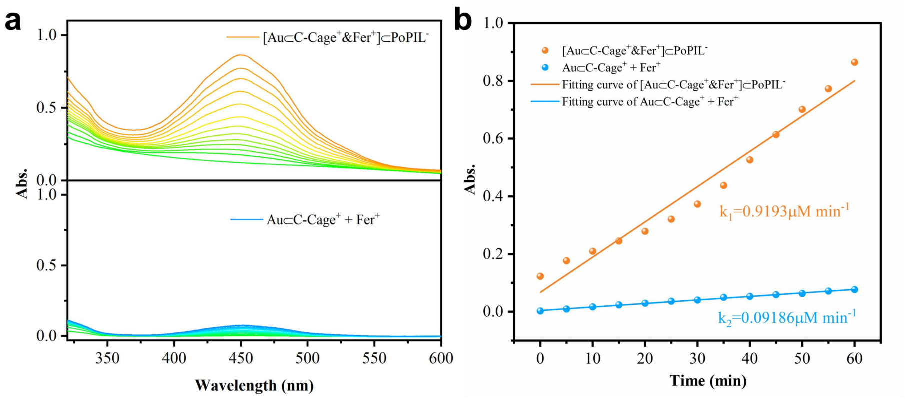

Supplementary Figure 61. (a) UV-Vis spectra and (b) linear fitting of reaction rate constant of the OPD oxidation cascade reaction by confined [Au⊂C-Cage$^+$&Fer$^+$]⊂PoPIL$^-$ catalyst and a mixture of free Au⊂C-Cage$^+$ and Fer$^+$ catalysts in the solution.

5. Supplementary Tables

Supplementary Table 1. Elemental analysis of the cage molecules and supramolecular assemblies

Entry	C (wt%)		H (wt%)		N (wt%)	
	**calcd.**	**found**	**calcd.**	**found**	**calcd.**	**found**
CC3	77.38	77.30	7.58	7.62	15.04	15.00
RCC3	75.74	75.70	9.53	9.55	14.72	14.66
C-Cage	54.76	54.66	7.66	7.58	10.64	10.73
CC3-OH	73.19	73.12	7.17	7.14	14.23	14.17
A-Cage	66.85	66.79	8.10	8.11	12.99	13.07
C-Cage-SS-12	60.19	60.08	6.13	6.05	5.01	5.17
C-Cage-SS-9	59.46	59.40	6.34	6.25	5.78	5.80
C-Cage-SS-6	58.45	58.39	6.62	6.50	6.82	6.93
C-Cage-VB	74.05	73.91	7.04	7.15	5.76	5.57
A-Cage-imidaz	69.09	69.09	7.89	7.94	19.34	19.42

Supplementary Table 2. Elemental analysis of the hypercrosslinked polymer composites

Entry	C (wt%)	H (wt%)	N (wt%)
C-Cage^{+}⊂PoPIL^{-}-12 (9:1) [a]	89.11	7.70	0.61
C-Cage^{+}⊂PoPIL^{-}-12 (3:1) [a]	82.53	7.42	1.53
C-Cage^{+}⊂PoPIL^{-}-12 (1:1) [a]	75.92	7.04	2.12
C-Cage^{+}⊂PoPIL^{-}-9 (1:1) [a]	76.10	7.12	2.74
C-Cage^{+}⊂PoPIL^{-}-6 (1:1) [a]	75.54	7.23	3.73
C-Cage^{+}⊂PoPIL^{-}-12 (1:1) [b]	83.01	7.29	2.53
A-Cage^{-}⊂PoPIL^{+}-4 (1:1) [c]	80.83	7.61	9.33
[Au⊂C-Cage^{+}]⊂PoPIL^{-} [d]	75.53	6.78	2.37
[Au⊂C-Cage^{+}&Fer^{+}]⊂PoPIL^{-} [d]	75.57	6.83	2.19

[a] SS as counteranion, the molar ratio of SS to C-Cage varies from 12:1 to 9:1 and 6:1, mass ratio of DVB to C-Cage-SS varies from 9:1 to 3:1 and 1:1.

[b] VB as counteranion, the molar ratio of VB to C-Cage is 12:1, mass ratio of DVB to C-Cage-VB is 1:1.

[c] Imidaz as countercation, the molar ratio of imidaz to A-Cage is 4:1, mass ratio of DVB to A-Cage-imidaz is 1:1.

[d] SS as counteranion, the molar ratio of SS to C-Cage is 12:1, mass ratio of DVB to C-Cage-SS is 1:1.

Supplementary Table 3. Surface areas and pore volume of the C-Cage^{+}⊂PoPIL^{-} and the A-cage^{-}⊂PoPIL^{+}

Entry	S_{BET} [a] (m^2/g)	S_L 1 (m^2/g)	S_{micro} [c] (m^2/g)	PV [d] (cm^3/g)
C-Cage^{+}⊂PoPIL^{-}-12 (9:1) [e]	836	1094	138	0.63
C-Cage^{+}⊂PoPIL^{-}-12 (3:1) [e]	647	1024	333	0.53
C-Cage^{+}⊂PoPIL^{-}-12 (1:1) [e]	410	726	212	0.43
C-Cage^{+}⊂PoPIL^{-}-9 (1:1) [e]	407	711	202	0.43
C-Cage^{+}⊂PoPIL^{-}-6 (1:1) [e]	418	715	207	0.44
C-Cage^{+}⊂PoPIL^{-}-12 (1:1) [f]	440	776	253	0.74
A-cage^{-}⊂PoPIL^{+}-4 (1:1) [g]	357	469	44	0.66

[a] Surface area calculated from N_2 sorption isotherms at 77 K using BET equation.

[b] Surface area calculated from N_2 sorption isotherms at 77 K using Langmuir equation.

[c] Microporous surface area calculated by t-plot method.

[d] Pore volume calculated from nitrogen isotherm at $P/P_0 = 0.995$, 77 K.

[e] SS as counteranion, the molar ratio of SS to C-Cage varies from 12:1 to 9:1 and 6:1, mass ratio of DVB to C-Cage-SS varies from 9:1 to 3:1 and 1:1.

[f] VB as counteranion, the molar ratio of VB to C-Cage is 12:1, mass ratio of DVB to C-Cage-VB is 1:1.

[g] Imidaz as countercation, the molar ratio of imidaz to A-Cage is 4:1, mass ratio of DVB to A-Cage-imidaz is 1:1.

Supplementary Table 4. Metal content determined by ICP-OES measurement

Entry	Metal	Content
Au⊂C-Cage$^+$	Au	2.42 wt%
[Au⊂C-Cage$^+$]⊂PoPIL$^-$	Au	0.78 wt%
Au⊂PoPIL$^-$	Au	0.47 wt%
[Au⊂C-Cage$^+$&Fer$^+$]⊂PoPIL$^-$	Au	0.74 wt%
	Fe	0.44 wt%
Aqueous solution after catalytic reaction ([Au⊂C-Cage$^+$]⊂PoPIL$^-$ catalyst was removed by centrifuge)	Au	< 5 ppm

Calculation of Debye screening length

Here, the concentrations of catalyst are considered to be negligible in comparison to the amount of $NaBH_4$ (10 mM) used in the reaction. The Debye screening lengths in different reaction media were calculated by using the following equation according to the literature [8, 9] :

$$\text{Supplementary Equation (6): } \kappa^{-1} = [\frac{N_a e^2}{\varepsilon_0 \varepsilon k_b T} \sum_i z_i^2 \, c_i^\infty]^{-1/2}$$

In this equation, κ^{-1} is defined as the Debye screening length, N_a is the Avogadro constant, e is the electron charge, ε is the dielectric constant of the media (80 for water) and ε_0 is the permittivity of the free space, k_b is Boltzmann constant and T is the temperature (in K), z_i is the valency of the ith ion and c_i^∞ is the concentration of ions of type i (in mol/m^3).

Using the equation, the Debye screening lengths for the reactions at different ionic strengths have been estimated and summarized in the table below.

Supplementary Table 5. Variation of Debye screening length with ionic strength of the reaction medium

	**Reaction in water**	**Reaction in 1X PBS**
Debye screening length (κ^{-1})	3.06 nm	0.76 nm

6. Computational Details

All simulations were performed with GROMACS 2020.4 package.[10] The models of Au cluster, inner C-Cage host, outer PoPIL⁻ shell and different charged substrates were produced with CGenFF,[11] which were then all placed in a cubic box with a side length of 8 nm generated by PACKMOL.[12] Subsequently, the models were solvated with water, and chloride ions and sodium ions were added to maintain charge neutrality. In these simulations, CHARMM 36 force field was used and the RESP charges were generated as follows: the molecules are optimized in B3LYP level using 6-31+G(d,p) basis set with Gaussian 09 program,[13] where the solvent effect is corrected by applying the SDM method. The RESP charges were properly optimized as well as the wave function by using Multiwfn.[14]

The cut-off for neighbor list of Verlet method and that for short-range interactions is 1.2 nm in all calculations with periodic boundary conditions in all three directions. The modified Berendsen thermostat (V-rescale in GROMACS) is used for temperature control. Berendsen pressure bath is used for possible pressure control. The Particle mesh Ewald (PME) method is used for electrostatics.

The catalysts were frozen in all systems and each system was subjected to energy minimization (with steepest descent algorithm), NVT and NPT equilibration phases (each 12 ns long), and final production phase (12 ns), from which the average density and other parameters were collected. Visualization of the simulations (figures and movies) were performed using Visual Molecular Dynamics (VMD). [15]

The distance between catalytic active center and substrates is expressed by the distance between Au cluster center and the S atom (in MO simulation) or N atom (in MB simulation), which can be used to evaluate the influence the affinity of substrate to catalyst. i.e., the closer distance between substrate and activate center, the easier for the substrate to be reacted.

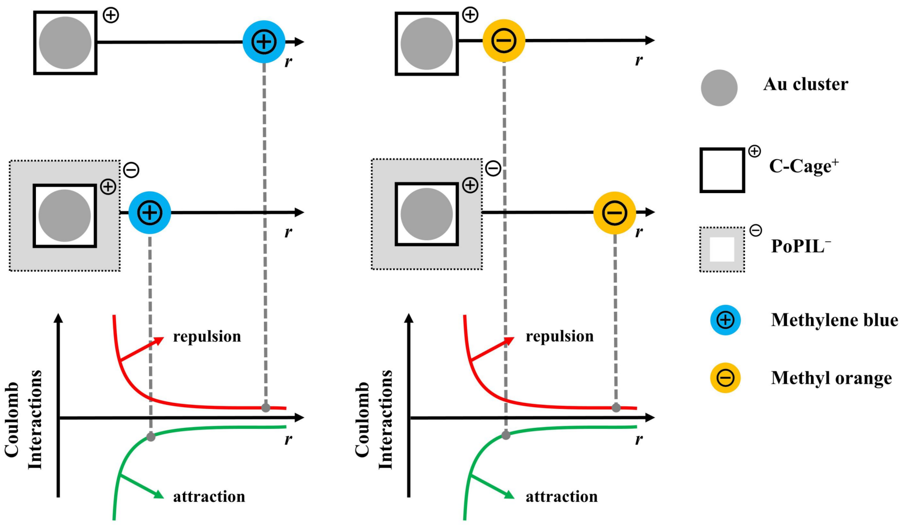

Supplementary Figure 62. Illustration for the comparation of Coulomb interactions between Au⊂C-Cage⁺ as well as [Au⊂C-Cage⁺]⊂PoPIL⁻ catalysts and different charged substrates (positive MB and negative MO).

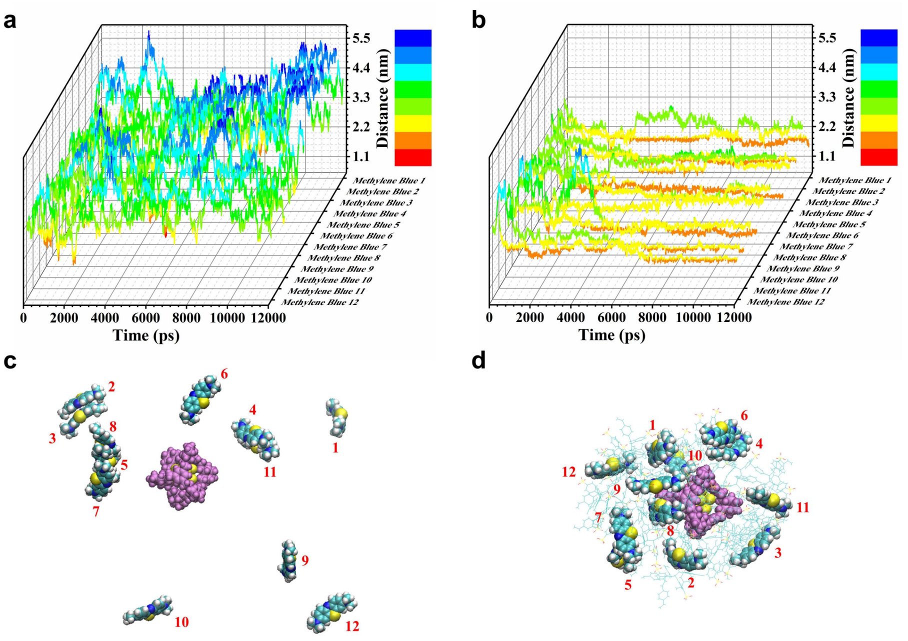

Supplementary Figure 63. Evolution of distance for the selected positive MB substrate (12 in total) during molecular dynamics (MD) process for (a) Au⊂C-Cage^{+} and (b) [Au⊂C-Cage^{+}]⊂PoPIL^{-} catalysts; and location of each MB substrates around (c) Au⊂C-Cage^{+} and (d) [Au⊂C-Cage^{+}]⊂PoPIL^{-} catalysts after kinetic equilibrium. For clarity, only the radial positions (with respect to Au cluster core) of N atoms in MB substrates are tracked during the MD simulation. Also see Supplementary Movie 1-2.

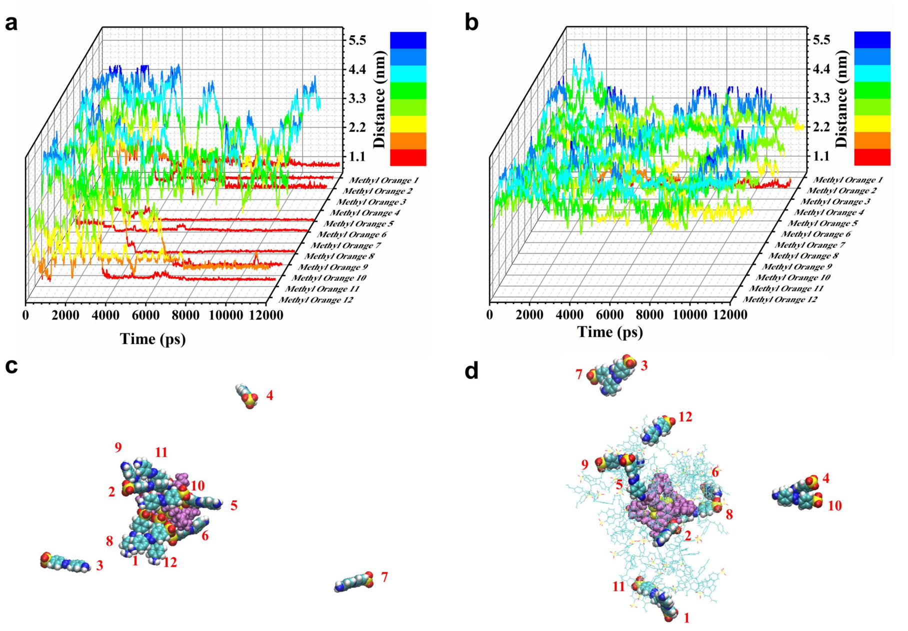

Supplementary Figure 64. Evolution of distance for each selected negative MO substrate (12 in total) during MD process for (a) Au⊂C-Cage^{+} and (b) [Au⊂C-Cage^{+}]⊂PoPIL^{-} catalysts; and location of each MO substrates around (c) Au⊂C-Cage^{+} and (d) [Au⊂C-Cage^{+}]⊂PoPIL^{-} catalysts after kinetic equilibrium. For clarity, only the radial positions (with respect to Au cluster core) of S atoms in MO substrates are tracked during the MD simulation. Also see Supplementary Movie 3-4.

In the MB MD simulation, the average distance of 2.11 nm between MB and [Au⊂C-Cage$^+$]⊂PoPIL$^-$ was closer than the average distance of 4.20 nm between MB and Au⊂C-Cage$^+$, indicating that the outer PoPIL$^-$ shell can attract MB substrates, and thus make it easier to be degraded by catalyst.

In the MO MD simulation, the average distance of 3.83 nm between MO and [Au⊂C-Cage$^+$]⊂PoPIL$^-$ was farther than the average distance of 1.46 nm between MO and Au⊂C-Cage$^+$, implying it is more difficult for the contact between [Au⊂C-Cage$^+$]⊂PoPIL$^-$ catalyst and MO substrate.

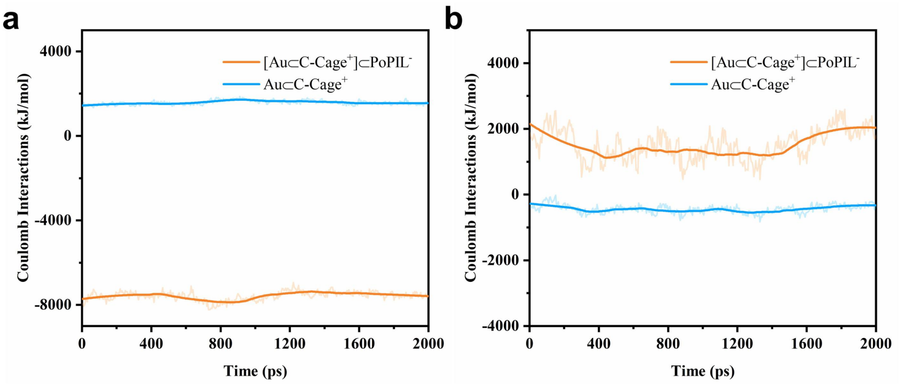

Supplementary Figure 65. Coulomb electrostatic interaction energy experienced by (a) MB and (b) MO substrate toward [Au⊂C-Cage$^+$]⊂PoPIL$^-$ and Au⊂C-Cage$^+$ catalysts. In all charts, light-color line represents the ‘raw’ data points, whereas the darker, solid lines mark the corresponding running averages.

Note for Supplementary Figure 65: The values for the Coulomb electrostatic interaction energy were computed using GROMACS tool, gmx energy. To reveal the role of PoPIL$^-$ in influencing the Coulomb electrostatic interactions between the catalyst and substrate, we first run the program to make the location of MB/MO substrates around [Au⊂C-Cage$^+$]⊂PoPIL$^-$ catalysts reach a kinetic equilibrium, and then analyze the Coulomb electrostatic interaction energy between the substrate and catalyst. Next, we constrained the location of the catalyst to the substrate, and remove the outer PoPIL$^-$ to analyze the Coulomb electrostatic interaction energy between Au⊂C-Cage$^+$ and substrate. By comparing the overall Coulomb electrostatic interaction energy between the catalyst and substrate with/without PoPIL$^-$, we can reveal the role of outer PoPIL$^-$ shell in driving the attraction/repulsion to the substrate with respect to the catalyst.

7. Supplementary References

1. Tozawa, T., et al. Porous organic cages. *Nat. Mater.* **8**, 973-978 (2009).

2. Zhang, S.-Y., et al. Ionic organic cage-encapsulating phase-transferable metal clusters. *Chem. Sci.* **10**, 1450-1456 (2019).

3. Liu, M., et al. Acid- and Base-Stable Porous Organic Cages: Shape Persistence and pH Stability via Post-synthetic "Tying" of a Flexible Amine Cage. *J. Am. Chem. Soc.* **136**, 7583-7586 (2014).

4. Liu, M., et al. Three-dimensional protonic conductivity in porous organic cage solids. *Nat. Commun.* **7**, 12750 (2016).

5. Chen, G.-J., Xin, W.-L., Wang, J.-S., Cheng, J.-Y.&Dong, Y.-B. Visible-light triggered selective reduction of nitroarenes to azo compounds catalysed by Ag@organic molecular cages. *Chem. Commun.* **55**, 3586-3589 (2019).

6. Xi, Z., et al. Nickel-Platinum Nanoparticles as Peroxidase Mimics with a Record High Catalytic Efficiency. *J. Am. Chem. Soc.* **143**, 2660-2664 (2021).

7. Li, S., et al. Label-free colorimetric detection of glutathione by autocatalytic oxidation of o-phenylenediamine based on Au^{3+} regulation and its application. *New J. Chem.* **45**, 9066-9072 (2021).

8. Roy, S., Rao, A., Devatha, G.&Pillai, P. P. Revealing the Role of Electrostatics in Gold-Nanoparticle-Catalyzed Reduction of Charged Substrates. *ACS Catal.* **7**, 7141-7145 (2017).

9. Walker, D. A., Wilmer, C. E., Kowalczyk, B., Bishop, K. J. M.&Grzybowski, B. A. Precision Assembly of Oppositely and Like-Charged Nanoobjects Mediated by Charge-Induced Dipole Interactions. *Nano Lett.* **10**, 2275-2280 (2010).

10. Van Der Spoel, D., Lindahl, E., Hess, B., Groenhof, G., Mark, A. E.&Berendsen, H. J. C. GROMACS: Fast, flexible, and free. *J. Comput. Chem.* **26**, 1701-1718 (2005).

11. Vanommeslaeghe, K., et al. CHARMM general force field: A force field for drug-like molecules compatible with the CHARMM all-atom additive biological force fields. *J. Comput. Chem.* **31**, 671-690 (2010).

12. Martínez, L., Andrade, R., Birgin, E. G.&Martínez, J. M. PACKMOL: A package for building initial configurations for molecular dynamics simulations. *J. Comput. Chem.* **30**, 2157-2164 (2009).

13. Frisch, M., et al. Gaussian 16. In. Gaussian, Inc. Wallingford, CT (2016).

14. Lu, T.&Chen, F. Multiwfn: A multifunctional wavefunction analyzer. *J. Comput. Chem.* **33**, 580-592 (2012).

15. Humphrey, W., Dalke, A.&Schulten, K. VMD: Visual molecular dynamics. *J. Mol. Graphics* **14**, 33-38 (1996).
