# Supplementary material for: Electrostatically cooperative host-in-host of metal cluster ⊂ ionic organic cages in nanopores for enhanced catalysis
Source: Nat Commun. 2022 Mar 18;13:1471. doi: 10.1038/s41467-022-29031-y (PMC8933400; doi:10.1038/s41467-022-29031-y)
Supplement: Supplementary file 3 — Description of Additional Supplementary Files [file 41467_2022_29031_MOESM3_ESM.pdf]

Description of Additional Supplementary Files

Supplementary Movie 1

MD simulation of Au⊂C-Cage$^+$ interacts with MB in water. The water molecules are omitted for a better view of the target molecules. The running time is 12 ns. Initially, MB molecule was placed at 3 nm away from the center of Au cluster. After 12 ns, the average distance of MB was stable in the system, was 4.20 nm.

Supplementary Movie 2

MD simulation of [Au⊂C-Cage$^+$]⊂PoPIL$^-$ interacts with MB in water. The water molecules are omitted for a better view of the target molecules. The running time is 12 ns. Initially, MB molecule was placed at 3 nm away from the center of Au cluster. After 12 ns, the average distance of MB was stable in the system, was 2.11 nm.

Supplementary Movie 3

MD simulation of Au⊂C-Cage$^+$ interacts with MO in water. The water molecules are omitted for a better view of the target molecules. The running time is 12 ns. Initially, MO molecule was placed at 3 nm away from the center of Au cluster. After 12 ns, the average distance of MO was stable in the system, was 1.46 nm.

Supplementary Movie 4

MD simulation of [Au⊂C-Cage$^+$]⊂PoPIL$^-$ interacts with MO in water. The water molecules are omitted for a better view of the target molecules. The running time is 12 ns. Initially, MO molecule was placed at 3 nm away from the center of Au cluster. After 12 ns, the average distance of MO was stable in the system, was 3.83 nm.
